# A novel study on the preferential attachment of chromophore and auxochrome groups in azo dye adsorption on different greenly synthesized magnetite nanoparticles: investigation of the influence of the mediating plant extract's acidity

**DOI:** 10.1039/d2na00302c

**Published:** 2022-07-13

**Authors:** Kaouthar Ahmouda, Moussa Boudiaf, Boubaker Benhaoua

**Affiliations:** Department of Process Engineering and Petrochemistry, Faculty of Technology, University of El Oued El Oued 39000 Algeria ahmouda-kaouthar@univ-eloued.dz drssmoussa@yahoo.fr; Renewable Energy Research Unit in Arid Zones, University of El Oued El Oued 39000 Algeria; LCIMN, Laboratory, Department of Process Engineering, Faculty of Technology, University Ferhat, Abbas Setif 19000 Sétif Algeria benhaouab@yahoo.fr; Department of Physics, Faculty of Exact Sciences, University of El Oued El Oued 39000 Algeria

## Abstract

In this paper, the adsorption of Evans blue (EB) and methyl orange (MO) azo dyes on four greenly synthesized magnetite nanoparticles has been studied to investigate the effect of the mediating plant extract's acidity on magnetite surface reactivity in azo dye adsorption. Magnetite surface reactivity has been studied through the analysis of preferential attachment of dye chromophore and auxochrome groups on magnetite nanoparticles, and adsorption yields. According to the contents of chromophore and auxochrome groups in dye structures, the mediating plant extract's acidity effect on acid site types and densities was also deduced. Used plants for the green synthesis were: *Artemisia herba-alba* (L), *Matricaria pubescens* (L), *Juniperus phoenicea* (L), and *Rosmarinus officinalis* (L), and their extract pHs were respectively 5.25, 5.05, 4.63, and 3.69. The four greenly synthesized samples of magnetite were characterized by XRD, SEM, ATR-FTIR, and UV-Vis techniques. The novelty of this paper lies in highlighting the influence of the mediating plant extract's acidity on the greenly synthesized magnetite surface reactivity towards the preferential attachment of chromophore and auxochrome functional groups in azo dye adsorption, where obtained results show that the mediating plant extract's acidity has a clear effect on the preferential attachment of chromophore and auxochrome groups on magnetite surfaces as well as on azo dyes' adsorption yields and capacities. Indeed, the decrease in the plant extract's acidity leads to an increase in the attachment of chromophore groups and a decrease in the attachment of auxochrome groups. So, it leads to an increase in Lewis acid site density and a decrease in Brønsted acid site density of magnetite surfaces. Also, the decrease of the plant extract's acidity leads to an increase in the studied dye adsorption yields, and this is because the majority of functional groups of MO and EB dyes are chromophores that attach to Lewis acid sites. The difference found in adsorption yields of EB and MO on all four magnetite samples is due to the fact that the ratio of chromophore/auxochrome groups in EB is remarkably greater than that in MO. The linear and non-linear pseudo-first-order and pseudo-second-order kinetics of the adsorption as well as the intra-particle diffusion mechanism have been analyzed. Obtained results indicate that in all adsorption processes the adsorption kinetics followed a linear pseudo-first-order kinetic model, and film diffusion is the step that controlled adsorption mechanisms. The thermodynamic studies of EB and MO adsorption processes on the four magnetite surfaces have been analyzed in the temperature range of 303.15–318.15 K. Obtained results reveal the endothermic nature of the adsorption in all cases.

## Abstract 2

The decrease in plant extract's acidity leads to the increase in Lewis acid site densities of greenly synthesized magnetite surfaces. The increase in Lewis acid site densities leads to the increase in azo dye adsorption yields.

## Graphical abstract

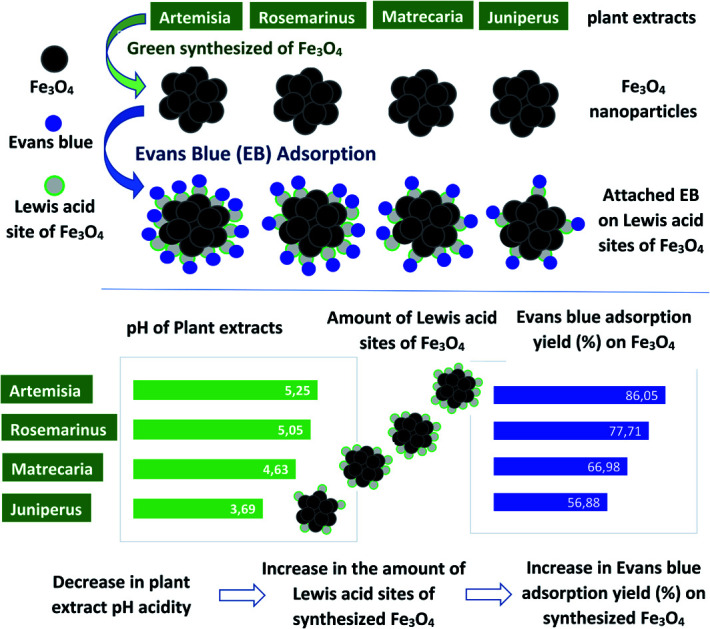

## 1 Introduction

Dyes are present in the effluent water of several industries, including textile, leather, paper, rubber, plastics, printing, cosmetics, pharmaceutical, and food industries. Dyes contribute to water toxicity and represent an increasing danger for the environment, humans, and animals.^1–3^ They are generally resistant to light, oxidizing agents, and many chemicals and therefore difficult to degrade once released into aquatic systems. Thus, one of the major environmental problems related to the numerous industrial applications of dyes is their removal from effluents.^3^

Dyes are composed of chromophores that are commonly electron withdrawing and auxochromes that are usually electron-releasing groups.^4^ The most important chromophores, as defined in this way, are: N=N, C=O, –CH=N, NO_2_, NO, NOH, C=N, C≡N, C=C, and C≡C groups and ionizing auxochromes mainly include: SO_3_H, OH, COOH, NH_2_, NH_3_, NHCH_3_, and N(CH_3_)_2_ groups.^4,5^

Adsorption is one of the most effective processes for the removal and recovery of colored materials and dyes from effluents.^6–9^ Nanomaterials are widely used in the purification of aqueous media due to their advantages, such as high surface area and increased number of active sites.^10–13^ They therefore allow a rapid thermodynamic equilibrium between adsorbent and adsorbate during the adsorption process and selective removal of pollutants.^7–9^ Several factors influence the adsorption process, mainly the solution chemistry,^7,14^ the characteristics of the dye (adsorbate)^7,15^ and the adsorbent surface properties.^16–22^ Saha *et al.*^7^ studied the preferential adsorption of seven different dyes on magnetite NPs. They reported that the magnetite surface preferred adsorbing dyes containing higher OH content. Xiao *et al.*^15^ studied the preferential adsorption of different cationic and anionic dyes on iron nanoparticles. They reported that iron NPs preferred removing cationic dyes more than anionic dyes.

Other authors studied the effect of changing the adsorbent surface properties by binding ligands on the adsorbent surface. Khurshid *et al.*^17^ found that the use of amine-functionalized cobalt–iron NP surface enhanced the removal of anionic azo dyes. Mahmoodi *et al.*^22^ synthesized a titania/silica nano-hybrid (TSNH) and an amine-functionalized titania/silica nano-hybrid (AFTSNH) to use them in Reactive Red 198 and Acid Red 14 removal from wastewater. They found that the AFTSNH adsorbent showed high dye adsorption capacities compared to the TSNH adsorbent. The authors of ref. 23 prepared silica nanoparticles (SN) and amine-functionalized silica nanoparticles (AFSN) and then used them in Acid Red 14, Acid Black 1 and Acid Blue 25 removal. They reported that AFSN preferred adsorbing the studied dyes than SN. Meanwhile, the study of Madrakian *et al.*^24^ reported that magnetite-modified activated carbon preferred adsorbing cationic dyes than anionic dyes. Moreover, a comparative study on adsorption of methylene blue on sericin-modified and unmodified magnetite NPs^25^ reported that sericin-modified magnetite NPs were approximately 40% more effective than the unmodified magnetite NPs. Furthermore, in the study of the preferential adsorption of magnetite NP loaded fig leaves (MNLFL) and magnetite NP loaded azolla (MNLA) to remove crystal violet and methylene blue,^26^ the authors found that MNLFL preferred adsorbing crystal violet more than MNLA.

Surface acid site types and densities were also found to impact the adsorption process. Indeed, a study of the adsorption of organic contaminants on both natural and synthesized magnetite^20^ found that the adsorption on natural magnetite was more efficient than that on the synthesized one, and this is because of its higher surface site density. Moreover, Gogoi *et al.*^27^ studied the degradation of catechol using an Fe_3_O_4_–CeO_2_ nanocomposite as a Fenton-like heterogeneous catalyst. They reported that the increase of Brønsted acid site density of this nanocomposite increased the degradation of catechol.

The impact of changing the mediating plants on the behavior of greenly synthesized metal oxide NPs in dye adsorption was studied in several works. The effect of three different tea extracts on the capacity of greenly synthesized iron oxide surfaces in the removal of methyl green dye from aqueous solution has been studied by Huang *et al.*^16^ They reported that the plant extract had an effect on the adsorption yields of methyl green dye on the three iron oxide NPs, where yields varied from 81.2% to 75.6 to 67.1%. Duyen *et al.*^28^ synthesized metal oxide NPs using the extracts of flowers, bark, and leaf of *Tecoma stans* in order to use them in the removal of Congo red (CR) and crystal violet (CV) dyes. They reported that the adsorbent derived from flower extract gave better adsorption efficiency than those derived from other extracts. Islam *et al.*^29^ synthesized magnetite NPs using six plant extracts in order to use them in the removal of methyl orange (MO) and crystal violet (CV) dyes. They reported that plant extract had an effect on magnetite NP surface reactivity in the adsorption, where magnetite NPs synthesized using tea extract showed the highest performance (MO 92.34%, CV 96.1%). Ahmouda *et al.*^30^ used different greenly synthesized magnetite NPs in the removal of methyl green (MG) dye *via* the adsorption process. They reported that the mediating plant extract's acidity had an effect on the preferential adsorption of MG on the magnetite NP surface. Indeed, magnetite NPs synthesized using plant extract having the lowest acidity exhibited the highest acid site density (OH groups) and hence the highest performance in the removal of MG.

As it is known that the adsorption of dyes from wastewater is directly affected by the reactivity of the adsorbent surface towards the attachment of dyes' functional groups, looking for parameters that could control the greenly synthesized magnetite NP surface reactivity is always of importance. In this way, this paper looks for mediating plant extract parameters that could, in the case of green synthesis, impact the magnetite NP surface reactivity in azo dye adsorption.

**Fig. 1:**
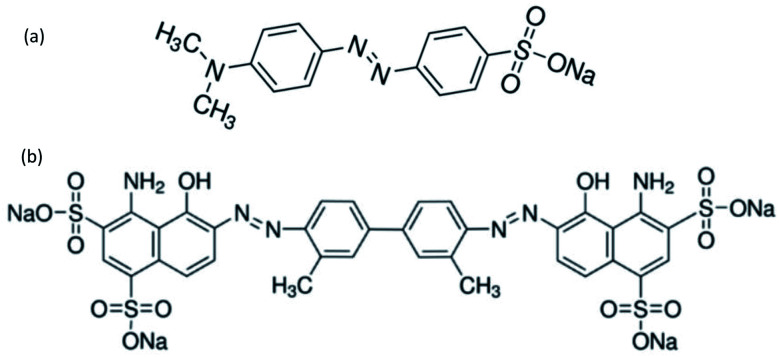
Chemical structures of (a) methyl orange and (b) Evans blue.

Azo dyes are the largest and most versatile class of organic dyes.^31^ These dyes contain one or more azo bonds (N=N). The complex aromatic structures of azo dyes make them more stable and more difficult to remove from the effluents discharged into water bodies.^31^ The used dyes in this study are: methyl orange (MO) and Evans blue (EB) azo dyes, and their chemical structures are illustrated in Fig. 1. The EB molecule is composed of chromophore groups such as benzene, phenyl, phenyldiazonium, toluene, N=N, C=C, and C=N groups linked to the benzene ring, and of auxochrome groups such as sulphonic acid (SO^−^_3_), phenol, and aniline groups. Meanwhile, the MO molecule is composed of chromophore groups such as benzene, phenyl, phenyldiazonium, N=N, C=C, and C=N, and of auxochrome groups such as sulphonic acid (SO^−^_3_) and dimethylamine (N(CH_3_)_2_).

The attachment of these functional groups on the adsorbent surface is based on functional group properties (chromophore or auxochrome) towards the surface acid site type (Lewis or Brønsted). In the case of EB and MO dyes, SO^−^_3_,^32^ dimethylamine,^33^ phenol,^34^ and aniline^35^ auxochrome groups are electron donating, and their attachment on the adsorbent surface is based on their ionic interaction with the developed positively charged Brønsted acid sites of the surface. Meanwhile, the chromophore groups phenyldiazonium, phenyl,^36^ benzene, N=N, C=C, C=N,^32^ and toluene^37^ are electron withdrawing, and their attachment on the adsorbent surface is based on their interactions with the Lewis acid sites of the adsorbent surface.

In this paper, the effect of the mediating plant extract's acidity on the greenly synthesized magnetite surface reactivity in the adsorption of methyl orange (MO) and Evans blue (EB) azo dyes has been investigated through the analysis of MO and EB adsorption on four greenly synthesized magnetite samples, with the aim of studying the effect of the mediating plant extract's acidity on the preferential attachment of the studied dye chromophores and auxochromes (functional groups) on greenly synthesized magnetite NPs, and adsorption yields. For this purpose, after the accomplishment of adsorption experiments, the free functional groups that are not attached on magnetite surfaces have been deeply analyzed in all dye residual solutions using ATR-FTIR spectroscopy, so as to perceive their preferential attachment on the four magnetite surfaces. Based on the analysis of preferential attachments of chromophore and auxochrome groups, it was possible to compare between Brønsted and Lewis acid site densities on each magnetite surface and their influence on adsorption yields and capacities. To the best of our knowledge, there is no study in the literature that has dealt with the influence of the mediating plant extract's acidity on magnetite surface reactivity towards the preferential attachment of dye functional groups, and thus on surface acid site type and density. The used plants are *Artemisia herba-alba* (L), *Matricaria pubescens* (L), *Juniperus phoenicea* (L), and *Rosmarinus officinalis* (L). Their extract pHs are respectively 5.25, 5.05, 4.63, and 3.69. The synthesized Fe_3_O_4_ samples are respectively denoted in this paper by ARM–Fe_3_O_4_, ROS–Fe_3_O_4_, MAT–Fe_3_O_4_ and JUN–Fe_3_O_4_. They were characterized by XRD, SEM, FTIR-ATR, and UV-Vis techniques.

## 2 Materials and methods

This section focuses on listing needed materials and used apparatuses. It also provides methods utilized to perform adsorption experiments and characterization of iron oxide NPs.

### 2.1 Materials

#### 2.1.1 Chemicals

Evans blue, methyl orange dyes, NaCl salt, HCl acid, and NaOH base are purchased from Sigma-Aldrich. JUN–Fe_3_O_4_, MAT–Fe_3_O_4_, ROS–Fe_3_O_4_ and ARM–Fe_3_O_4_ nanoparticle powders were greenly synthesised using iron salt (FeCl_3_·6H_2_O) (purchased from Biochem Chemopharma Co, Canada) as precursor and *Artemisia herba-alba* (L) (Asteraceae family), *Matricaria pubescens* (L) (Asteraceae family), *Juniperus phoenicea* (L) (Cupressaceae family), and *Rosmarinus officinalis* (L) (Lamiaceae family) plants as reducing agents. Magnetite samples were obtained after 4 months of storage of the synthesized iron oxides under ambient conditions. Characterization of the freshly synthesized iron oxides samples is presented in Ahmouda *et al.*^38^

#### 2.1.2 Apparatuses

XPERT-PRO X-ray diffractometer (XRD) (Rigaku Miniflex 600) with 30 keV and 30 mA as conditions of X-ray generation and Kα radiation of copper (*λ* = 1.54056 Å). Attenuated total reflectance-Fourier transform infrared spectroscopy (ATR-FTIR): Shimadzu IR-Infinity. Ultraviolet-visible spectroscopy (UV-Vis): Shimadzu UV-Vis spectrophotometer apparatus Model 1800 operating in the range of 200–900 nm. SEM-TESCAN VEGA3 Model XMU, LMH (Brno, Czech Republic) for speeding up the voltage of 15–20 kV in order to capture the scanning electron micrograph (SEM) of greenly synthesised iron oxide NPs.

### 2.2 Methods

In this section, used methods for solution preparation are described. Used protocols in adsorption and desorption experiments of dyes from magnetite NPs and characterization techniques are described as well.

#### 2.2.1 Batch adsorption experiments of MO and EB dyes

First, the prepared standard aqueous solutions of EB and MO dyes were diluted several times as required. Then, 0.0015 g of JUN–Fe_3_O_4_, MAT–Fe_3_O_4_, ROS–Fe_3_O_4_ and ARM–Fe_3_O_4_ powders were added to a volume of 4 mL of dye aqueous solutions. The dye solution concentration was 0.0111 mg mL^−1^. The ionic strength for all adsorption experiments was kept at 0.1 M by adding an appropriate amount of NaCl (0.023 g). A dilute solution of HCl was used to adjust the dye/Fe_3_O_4_ solution pH to 4. This protocol is used to prepare, in total, 88 experiment sets (11 with each dye/magnetite sample). In addition, control experiment sets (without NPs) were also prepared.

All experiment sets are sonicated in an ultrasonic bath for 15 minutes and they were then stirred continually for 60 minutes until a steady state was reached. All adsorption experiments were carried out under ambient conditions in batch mode, and they were performed in triplicate for data consistency.

Kinetic experiments were performed by withdrawing samples of dye/Fe_3_O_4_ solutions at a regular time interval to obtain, after centrifugation, adequate aliquots for the purpose of quantifying residual dye concentrations and the adsorbed amounts. The concentrations of residual dye aqueous solutions were quantified using a UV-Vis spectrophotometer at absorbance maxima of EB (*λ*_max_ = 602 nm) and MO (*λ*_max_ = 463 nm). Furthermore, the adsorbed amounts of EB and MO molecules are calculated from the calibration curve for all adsorption experiments (*Y* = 67.02*X* + 0.0442, *R*^2^ = 0.9987 and *Y* = 31.39*X* + 0.0346, *R*^2^ = 0.9985, respectively). On another side, after the adsorption was accomplished and steady state reached, the aliquots were centrifuged to separate liquid solutions and solid phases. The liquid solutions, which represent MO/magnetite and EB/magnetite residual solutions, were then analyzed using ATR-FTIR spectroscopy.

To calculate the adsorption capacity (*q*_e_ in mg g^−1^) and the amount of MO and EB ions adsorbed per unit mass (*q*_*t*_ in mg g^−1^) of JUN–Fe_3_O_4_, MAT–Fe_3_O_4_, ROS–Fe_3_O_4_ and ARM–Fe_3_O_4_ at equilibrium contact time, the following equations were used:1
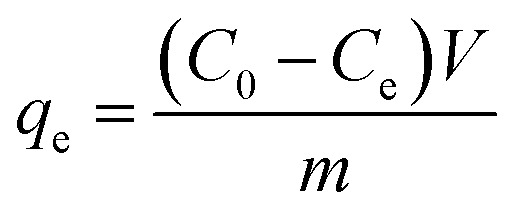
2
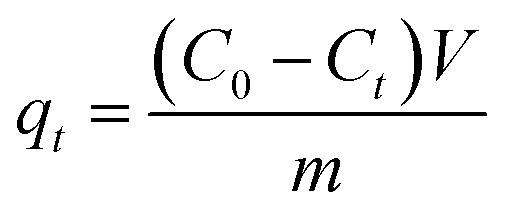


Adsorption yield was calculated using the following equation:3
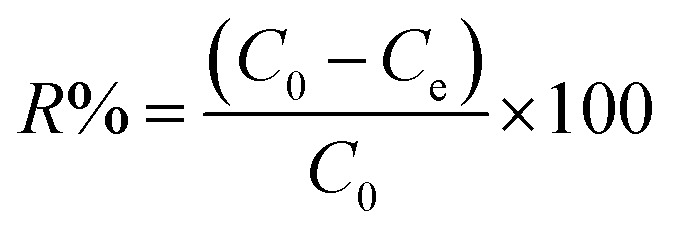
where *C*_0_, *C*_e_, *C*_*t*_, *V* and *m* are respectively: initial dye concentration in the liquid phase (mg mL^−1^), liquid phase dye concentration at the steady state (mg mL^−1^), liquid phase dye concentration at time *t* (mg mL^−1^), volume of dye solution used (mL), and the amount of adsorbent (g).

#### 2.2.2 Linear and non-linear pseudo-first-order and pseudo-second-order kinetics

The linear (eqn (4)) and non-linear (eqn (5)) pseudo-first-order (PFO) or Lagergren,^39^ and linear (eqn (6)) and non-linear (eqn (7)) pseudo-second-order (PSO) or Ho and McKay^40^ kinetic models are selected to test the adsorption dynamics in this study due to their good applicability in most studies.^41–43^

4 ln(*q*_e_ − *q*_*t*_) = ln *q*_e_ − *K*_1_*t*

5 *q*_*t*_ = *q*_e_(1 − e^−*K*_1_*t*^)

where *K*_1_, *q*_*t*_ and *q*_e_ are respectively: the pseudo first order kinetic constant (min^−1^), adsorbed dye quantity at instant *t* (mg g^−1^) and adsorbed dye quantity at thermodynamic equilibrium (mg g^−1^).

If the active surface of the adsorbent is regarded as invariable, the reaction could be treated as pseudo-first-order kinetic. However, once the active sites have been saturated, the transfer at the adsorbate/adsorbent particle interface may be limited by mass transfer.^44^

The pseudo second-order (PSO) model is proposed by Ho and McKay.^40^ It is based on the adsorption capacity expressed as follows:6
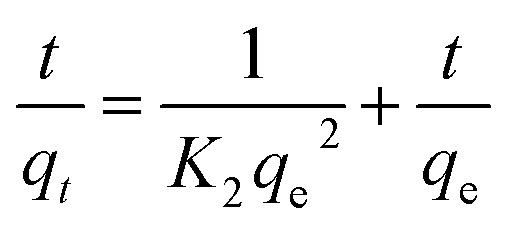
7
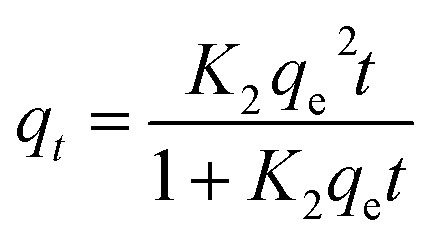
where *K*_2_, *q*_*t*_ and *q*_e_ are the pseudo second order kinetic constant (mg g^−1^ min^−1^), adsorbed dye quantity at instant *t* (mg g^−1^) and adsorbed dye quantity at thermodynamic equilibrium (mg g^−1^), respectively.

#### 2.2.3 Intra-particle diffusion kinetics

In order to gain insights into the adsorption mechanisms involved, a homogeneous particle diffusion model (HPDM) as shown in eqn (8), originally proposed by Boyd *et al.*,^45^ is used to describe the diffusive adsorption process. In this model, the rate-limiting step is usually described by either an intra-particle diffusion or a film diffusion mechanism.8
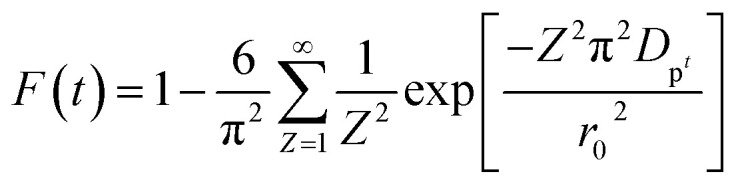
where *F*(*t*) is the fractional attainment at time *t*, *i.e.*, *F*(*t*) = *q*_*t*_/*q*_e_, *D*_p_ (m^2^ s^−1^) is the effective diffusion coefficient, *r*_0_ is the radius of Fe_3_O_4_ particles assumed to be spherical, and *Z* is an integer. For 0 < *F*(*t*) < 1, a simplified equation can be obtained for the adsorption on spherical particles:9
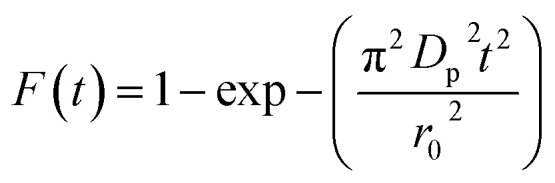


A further formula manipulation gives the following:10
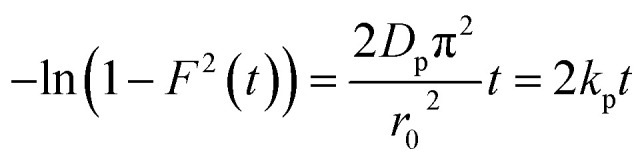
where *k*_p_ is the diffusion rate constant (1/*s*) and *k*_p_ = *D*_p_π^2^/*r*^2^_0_. Eqn (10) is used for the calculation of effective intra-particle diffusivity (*D*_p_ (m^2^ s^−1^)) from the experimental data. In the first step, a graph of −ln(1 − *F*^2^(*t*)) *vs. t* is developed. The values of *k*_p_ of EB and MO for JUN–Fe_3_O_4_, MAT–Fe_3_O_4_, ROS–Fe_3_O_4_ and ARM–Fe_3_O_4_ adsorption processes are obtained from the slopes of the fitted lines (plots of −ln(1 − *F*^2^) *vs.* time), and the values of effective diffusion coefficient, *D*_p_ (m^2^ s^−1^), can then be obtained from *D*_p_ = *k*_p_*r*^2^_0_/π^2^.

Additionally, eqn (11) can be used when the rate of adsorption is controlled by liquid film diffusion.^46^11
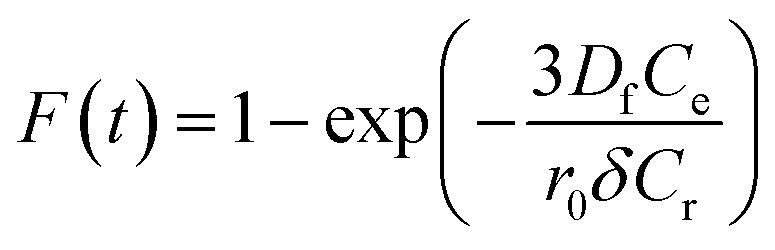
where *D*_f_ is the film diffusion coefficient (m^2^ s^−1^) in the liquid phase, and *C*_e_ (mol L^−1^) and *C*_r_ (mol L^−1^) are respectively the equilibrium concentrations of dye in solution and in solid phases. *δ* is the thickness of the liquid film which was assumed to be 10^−5^ m according to Yu and Luo.^47^ A further formula deformation of eqn (11) gives the following equation:

12 −ln(1 − *F*) = *k*_f_*t*

where *k*_f_ is the diffusion rate constant (1/*s*).

The values of *k*_f_ = 3*D*_f_*C*_e_/*r*_0_*δC*_r_ for the adsorption of MO and EB on ARM–Fe_3_O_4_, ROS–Fe_3_O_4_, MAT–Fe_3_O_4_, and JUN–Fe_3_O_4_ are obtained from the slopes of the fitted lines (plots of −ln(1 − *F*) *vs.* time), and the values of effective diffusion coefficient, *D*_f_ (m^2^ s^−1^), can then be obtained from *D*_f_ = *k*_f_*r*_0_*C*_r_/3*C*_e_.

The linearity test of Boyd plots −ln(1 − *F*) and −ln(1 − *F*^2^) *versus* time plots is employed to distinguish between the film diffusion and particle diffusion-controlled adsorption mechanism. If the plot of −ln(1 − *F*) *versus* time is a straight line passing through the origin, then the adsorption rate is governed by the particle diffusion mechanism, otherwise if −ln(1 − *F*^2^) *versus* time is a straight line passing through the origin then the adsorption is governed by film diffusion.

#### 2.2.4 Batch desorption experiments of MO and EB dyes

In order to regenerate used magnetite samples after the end of dye adsorption, a dilute solution of NaOH was used to adjust the dye/Fe_3_O_4_ solution pH in the range of 8–12. All experiment sets were sonicated in an ultrasonic bath for 15 minutes and they were then stirred continually for 60 minutes. All desorption experiments were carried out in ambient conditions in batch mode, and they were performed in triplicate for data consistency. This protocol is used to regenerate, in total, 8 magnetite samples. In addition, control experiment sets (without NPs) were also prepared. After centrifugation, adequate aliquots were taken for the purpose of quantifying desorbed dye concentrations and the desorbed amounts from magnetite surfaces. The concentrations of desorbed dye from magnetite surfaces were quantified using a UV-Vis spectrophotometer at absorbance maxima *λ*_max_ = 602 nm for EB and *λ*_max_ = 463 nm for MO. Furthermore, the desorbed amounts of EB and MO molecules are calculated from the calibration curve for all adsorption experiments (*Y* = 67.02*X* + 0.0442, *R*^2^ = 0.9987 and *Y* = 31.39*X* + 0.0346, *R*^2^ = 0.9985, respectively).

To calculate the desorption yields (*R*%) of MO and EB ions at contact time *t* = 60 min from JUN–Fe_3_O_4_, MAT–Fe_3_O_4_, ROS–Fe_3_O_4_ and ARM–Fe_3_O_4_ surfaces, the following equation was used:13
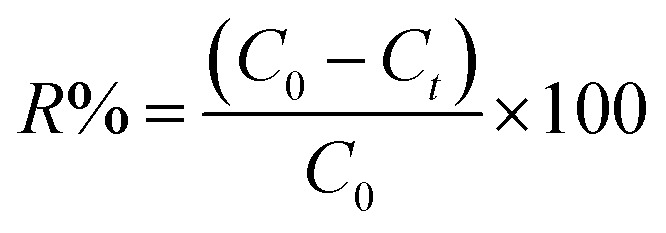
where *C*_0_ and *C*_*t*_ are respectively: initial dye concentration adsorbed on the solid phase (magnetite) (mg mL^−1^) and liquid phase desorbed dye concentration at contact time *t* = 60 min (mg mL^−1^).

#### 2.2.5 Thermodynamic studies of EB and MO adsorption processes on magnetite surfaces

In order to study the thermodynamics of EB and MO adsorption processes, all sets of experiments are sonicated in an ultrasonic bath for 15 minutes. Then, they are stirred continually for 20 minutes at temperatures ranging from 303.15 to 318.15 K. The concentrations of residual EB and MO dyes in liquid phases are quantified using a UV-Vis spectrophotometer at absorbance maxima *λ*_max_ = 602 nm for EB and *λ*_max_ = 463 nm for MO. Furthermore, the adsorbed amounts of EB and MO molecules are calculated from the calibration curves for all adsorption experiments (*Y* = 67.02*X* + 0.0442, *R*^2^ = 0.9987 and *Y* = 31.39*X* + 0.0346, *R*^2^ = 0.9985, respectively).

The adsorption capacity, *q*_e*T*_ (mg g^−1^), was calculated using the following equations:14
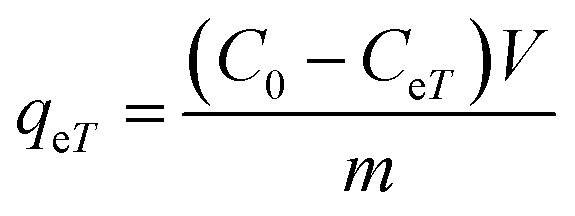


Adsorption yield was calculated using the following equation:15
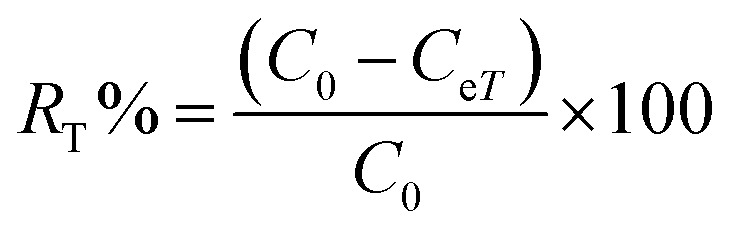
where *C*_0_, *C*_e*T*_, *V* and *m* are respectively: initial dye concentration without any treatment (mg mL^−1^), residual dye concentration in the liquid phase (mg mL^−1^) at a given temperature *T*, the volume of dye solution (mL), and the amount of magnetite NPs (g).

The activation enthalpy (Δ*H*^0^) of EB and MO adsorption processes on the magnetite NP surface was determined using the Arrhenius equation as follows:16
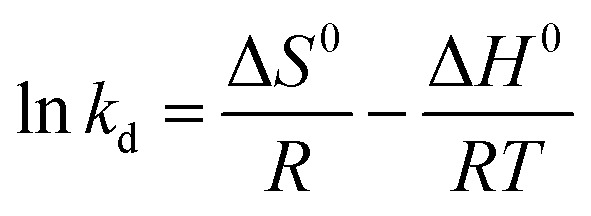
where *R* (1.987 cal mol^−1^ K^−1^) is the universal gas constant, *T* is the absolute solution temperature (K), and *k*_d_ is the distribution coefficient which was calculated by:17
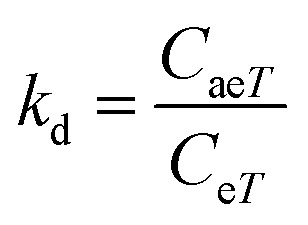
where *C*_ae*T*_ (mg mL^−1^) and *C*_e*T*_ (mg mL^−1^) are respectively the concentration of adsorbed dye on the solid (magnetite) and dye residual concentration in the liquid phase at a given temperature *T*.

The values of activation enthalpy Δ*H*^0^ (kcal mol^−1^) and entropy Δ*S*^0^ (cal mol^−1^ K^−1^) were respectively calculated from the slope and intercept of plots between ln *k*_d_ and 1/*T*. Δ*G*^0^ (kcal mol^−1^) was then calculated using the following equation:

18 Δ*G*^0^ = −*RT* ln *k*_d_

The free energy change indicates the degree of spontaneity of the adsorption process. The higher negative value reflects more energetically favorable adsorption. The activation energy, Δ*E*_a_ (kcal mol^−1^), of EB and MO adsorption processes on magnetite surfaces was determined using Arrhenius's equation:19
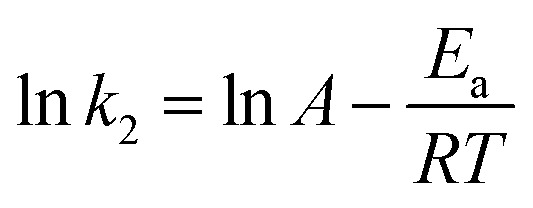
where *k*_2_ is the distribution coefficient which can be calculated by:20
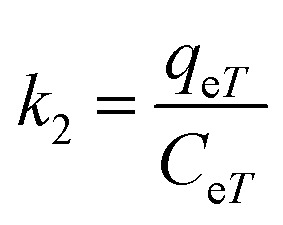
where *q*_e*T*_ (mg g^−1^) and *C*_e*T*_ (mg mL^−1^) are respectively the adsorption capacity of dye on the solid and dye residual concentration in the liquid phase at temperature *T*.

## 3 Results and discussion

### 3.1 X-ray analysis of magnetite nanoparticle samples

**Fig. 2:**
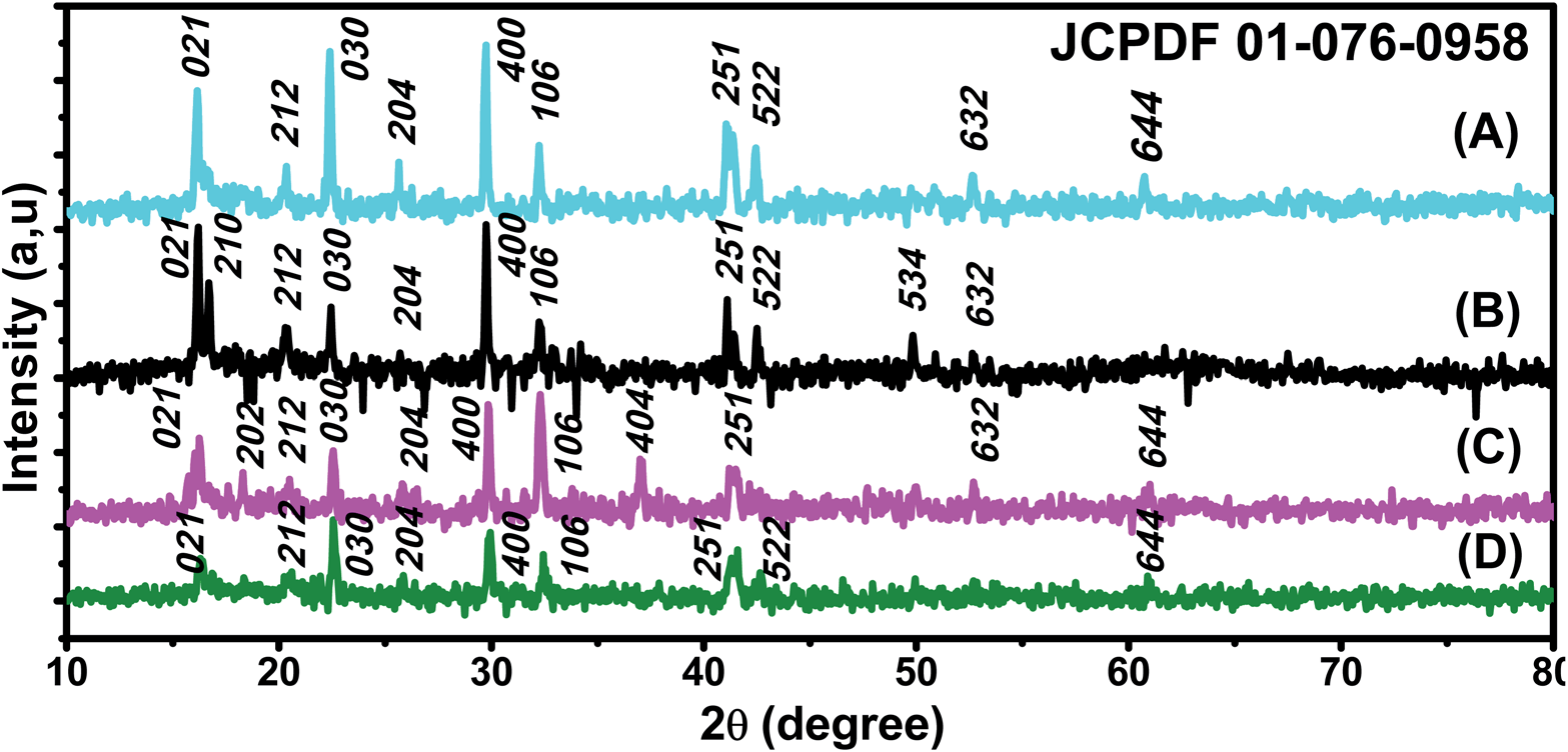
XRD patterns of (A) ROS–Fe_3_O_4_, (B) ARM–Fe_3_O_4_, (C) MAT–Fe_3_O_4_, and (D) JUN–Fe_3_O_4_ NPs (JCPDS file 01-076-0958).

X-ray patterns of all synthesised samples are presented in Fig. 2. It is found that all synthesized samples have crystalline structures. The X-ray diffraction pattern in Fig. 2(A) exhibits Bragg reflection peaks at around 2*θ* = 16.20°, 20.30°, 22.39°, 25.60°, 29.72°, 32.30°, 41.05°, 41.39°, 42.48°, and 52.69°. All Bragg peaks are in agreement with orthorhombic Fe_3_O_4_ powder and correspond to Miller indices 021, 212, 030, 400, 314, 001, 250, 251, 522, and 644, respectively (JCPDS file 01-076-0958).

The X-ray diffraction pattern in Fig. 2(B) exhibits Bragg reflection peaks at around 2*θ* = 16.20°, 16.70°, 20.39°, 22.42°, 29.75°, 30.80°, 32.30°, 41.10°, 42.53°, 49.82°, and 52.72°. All Bragg peaks are in agreement with orthorhombic Fe_3_O_4_ powder and correspond to Miller indices 021, 210, 212, 030, 400, 041, 106, 251, 522, 534, and 644, respectively (JCPDS file 01-076-0958).

The X-ray diffraction pattern in Fig. 2(C) exhibits Bragg reflection peaks at around 2*θ* = 16.20°, 22.56°, 26.04°, 32.28°, 37.11°, 41.59°, 49.98°, and 52.69°. All Bragg peaks are in agreement with orthorhombic Fe_3_O_4_ powder and correspond to Miller indices 021, 030, 400, 106, 404, 251, 534, and 644, respectively (JCPDS file 01-076-0958).

The X-ray diffraction pattern in Fig. 2(D) exhibits Bragg reflection peaks at around 2*θ* = 16.35°, 20.58°, 22.60°, 25.77°, 29.94°, 32.47°, 41.59°, 42.69°, 49.98°, and 52.69°. All Bragg peaks are in agreement with orthorhombic Fe_3_O_4_ powder and correspond to Miller indices 021, 212, 030, 400, 001, 106, 251, 522, 534, and 644, respectively (JCPDS file 01-076-0958).

The average diameters of different magnetite samples, presented in Table 1, are calculated from XRD patterns using Scherrer's equation:^48^21
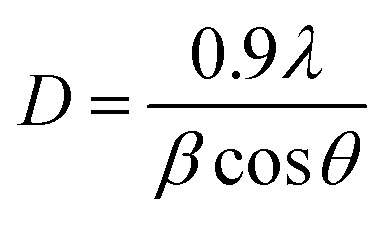
where *D*, *β*, *λ*, and *θ* are the crystallite size, the full width at half-maximum (FWHM) of the most intense diffraction peak, the X-ray wavelength (1.54056 Å), and Bragg angle, respectively.

**Table 1:** Calculated average diameters of ROS–Fe_3_O_4_, ARM–Fe_3_O_4_, MAT–Fe_3_O_4_, and JUN–Fe_3_O_4_ NPs

Samples	Average diameter (nm)
ARM–Fe_3_O_4_	41.49
ROS–Fe_3_O_4_	39.89
MAT–Fe_3_O_4_	33.13
JUN–Fe_3_O_4_	29.27

### 3.2 ATR-FTIR spectroscopy analysis

**Fig. 3:**
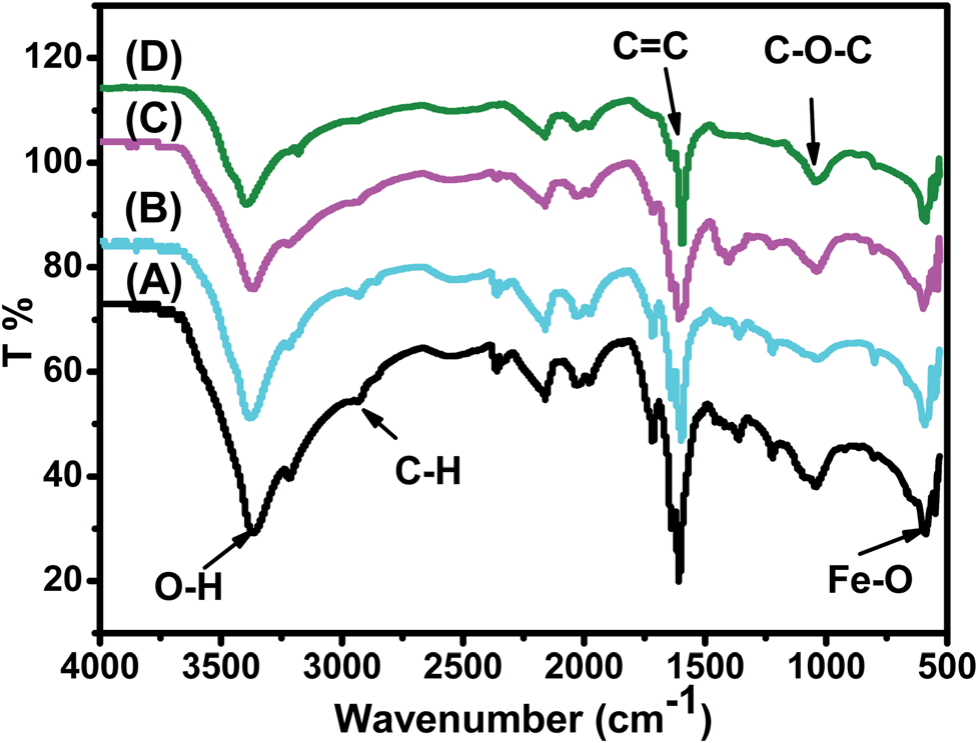
FTIR spectra of the synthesized (A) ARM–Fe_3_O_4_, (B) ROS–Fe_3_O_4_, (C) MAT–Fe_3_O_4_, and (D) JUN–Fe_3_O_4_ samples.

FTIR spectra of the synthesized Fe_3_O_4_ nanoparticle powders recorded between 4000 and 500 cm^−1^ are presented in Fig. 3. Fig. 3 shows that all IR spectra (A, B, C, and D) exhibit peaks in different ranges as is summarized in Table 2. The peaks at 3223.41–3266.69 cm^−1^ correspond to the O–H stretching vibration, while the peaks at 2930.18–2932.06 cm^−1^ correspond to C–H vibrations. The peaks at 1590.07–1594.63 cm^−1^ correspond to C=C stretching in aromatic rings and anti-symmetric stretching of the carboxylate group (COO^−^), whereas peaks at 1033.45–1044.36 cm^−1^ are assigned to the symmetric stretching vibration of the C–O–C functional group of the phenolic groups.^49^ The peak at around 592 cm^−1^ corresponds to the Fe–O stretching band of Fe_3_O_4_ NPs.^50^

**Table 2:** FTIR vibrations of the Fe_3_O_4_ functional group

Sample	O–H cm^−1^	C–H cm^−1^	C=C cm^−1^	C–O–C cm^−1^	Fe–O cm^−1^
ARM–Fe_3_O_4_	3266.69	2932.06	1590.07	1036.36	592.64
ROS–Fe_3_O_4_	3249.77	2930.18	1590.83	1038.75	591.83
MAT–Fe_3_O_4_	3235.57	2929.75	1591.21	1039.54	592.46
JUN–Fe_3_O_4_	3223.41	2928.82	1594.63	1039.45	592.69

Fig. 3 shows that the peaks of hydroxyl groups appear in remarkably different areas. Meanwhile, the hydroxyl group peak area appears to be the broadest on the ARM–Fe_3_O_4_ surface, next on ROS–Fe_3_O_4_, then on MAT–Fe_3_O_4_, and finally on JUN–Fe_3_O_4_. This reveals that the density of functional OH groups is higher on the ARM–Fe_3_O_4_ surface, next on ROS–Fe_3_O_4_, then on MAT–Fe_3_O_4_, and finally on JUN–Fe_3_O_4_.

### 3.3 UV-vis spectroscopy analysis

The optical absorbance spectra of all the synthesized Fe_3_O_4_ are measured in the wavelength range of 200–900 nm, to deduce their band gap energies. The band gap *E*_g_ and the optical absorption coefficient (*α*) of a direct band gap semiconductor are related through the known following equation:^51^

22 *αhν* = *A*(*hν* − *E*_g_)^*n*^

where *α* is the linear absorption coefficient of the material, *hν* is the photon energy, *A* is a proportionality constant, and the exponent *n* depends on the nature of electronic transition; it is equal to 1/2 for a direct allowed transition and 2 for an indirect allowed transition.

**Fig. 4:**
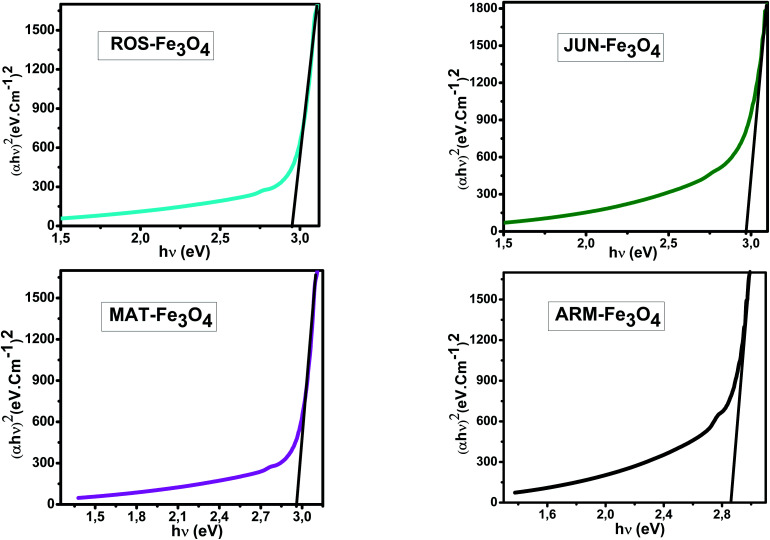
Plots of (*αhν*)^2^*versus* (*αhν*) for direct transition of synthesized Fe_3_O_4_ samples sonicated in acetone for 15 minutes.

**Fig. 5:**
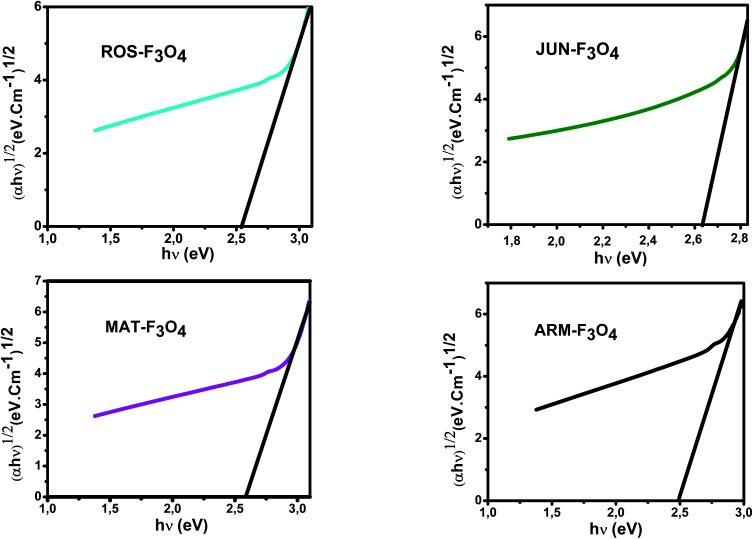
Plots of (*αhν*)^1/2^*versus* (*αhν*) for indirect transition of synthesized Fe_3_O_4_ samples sonicated in acetone for 15 minutes.

*E*_g_ of the direct transition of all samples were obtained from plotting (*αhν*)^2^ as a function of *αhν* by the extrapolation of the linear portion of the curve (Fig. 4). However, *E*_g_ of the indirect transition of all samples were obtained from plotting (*αhν*)^1/2^ as a function of *αhν* by the extrapolation of the linear portion of the curve (Fig. 5).

Estimated direct band gap energies of JUN–Fe_3_O_4_, MAT–Fe_3_O_4_, ROS–Fe_3_O_4_, and ARM–Fe_3_O_4_ samples were found to be 2.97, 2.96, 2.95 and 2.87 eV, respectively, which are close to that found by El Ghandoor *et al.*^52^ They found that direct gap energy for Fe_3_O_4_ equals *E*_g_ = 2.87 eV.

The estimated indirect band gap energies of ARM–Fe_3_O_4_, ROS–Fe_3_O_4_, MAT–Fe_3_O_4_ and JUN–Fe_3_O_4_ phases were found to be 2.51, 2.55, 2.60 and 2.64 eV, respectively, which are higher than reported by other authors.^52^ They found that indirect gap energy for Fe_3_O_4_ equals *E*_g_ = 1.92 eV.

It is clear that the direct gap energy is closer to the theoretical value than the indirect gap energy. The values of all direct band gap energies of magnetite samples classify them as semiconductors. The energy band gap of the semiconductors is between 0 and 3 eV.^53^

### 3.4 SEM images of the plant–Fe_3_O_4_ samples

**Fig. 6:**
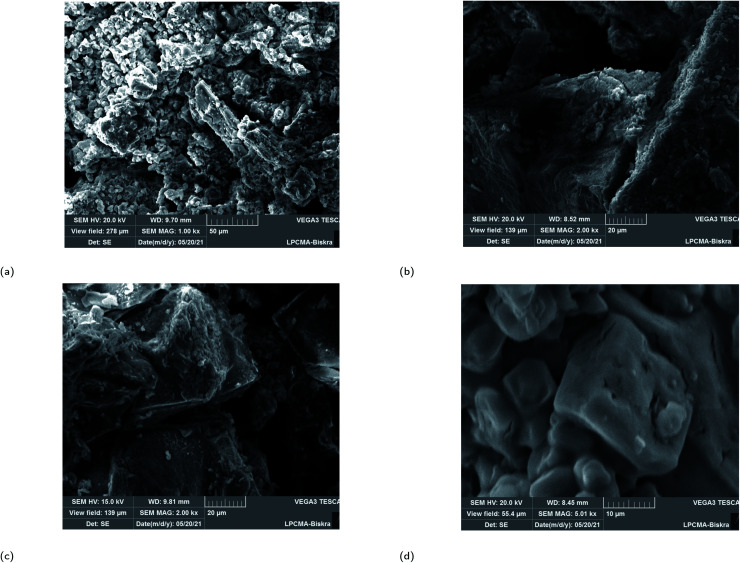
SEM images of greenly synthesized (a) JUN–Fe_3_O_4_, (b) MAT–Fe_3_O_4_, (c) ROS–Fe_3_O_4_ and (d) ARM–Fe_3_O_4_ NPs.

SEM images of the plant–Fe_3_O_4_ samples are presented in Fig. 6. It is clearly shown that the morphology of all four magnetite NPs depends on the plant extract. Different irregular rock-like shapes are observed in all samples. For ROS–Fe_3_O_4_ NPs, it is clear that a few agglomerations like rocks are present as shown in Fig. 6a. Meanwhile, for JUN–Fe_3_O_4_ NPs, bigger rocks that look like mountains are visible, as shown in Fig. 6b. However, for MAT–Fe_3_O_4_ a decrease in the dimensions of the mountains with more adherence to its structure is observed (Fig. 6c). Finally, the ARM–Fe_3_O_4_ SEM image sometimes contains large-structured single bipyramid crystal as mentioned in Fig. 6d.

### 3.5 Adsorption experiment equilibrium, kinetics analysis, and thermodynamic study

#### 3.5.1 Adsorption experiment equilibrium

**Fig. 7:**
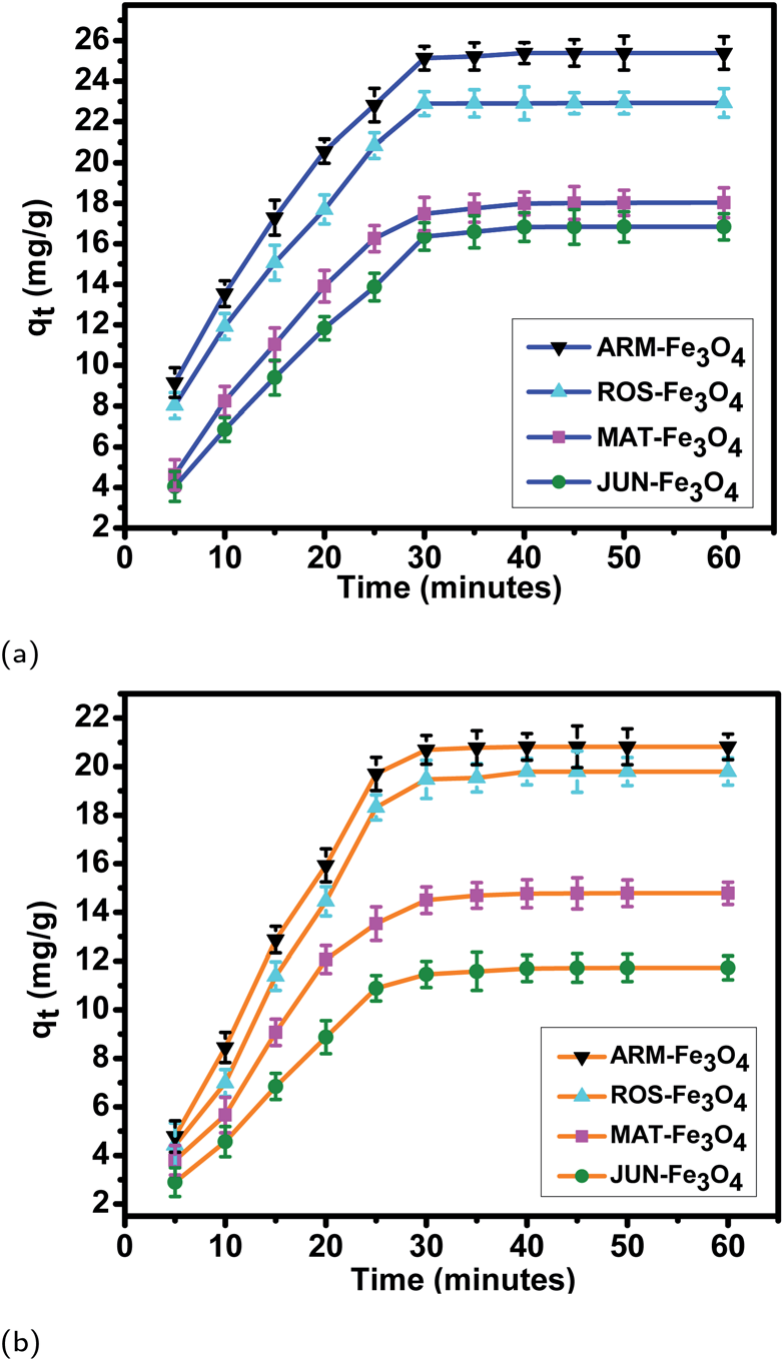
Adsorption capacities of (a) EB and (b) MO on ARM–Fe_3_O_4_, ROS–Fe_3_O_4_, MAT–Fe_3_O_4_, and JUN–Fe_3_O_4_ surfaces. Error bars represent the standard deviation of three replicates.

In all adsorption experiments, the steady state is reached within 30 minutes, as depicted in Fig. 7, and Tables 3 and 4. This denotes a very fast adsorption kinetics of MO and EB dyes on ARM–Fe_3_O_4_, ROS–Fe_3_O_4_, MAT–Fe_3_O_4_ and JUN–Fe_3_O_4_ surfaces. The fast initial adsorption is mainly due to the rapid attachment of dye molecules to favorable active sites of magnetite surfaces through mass transfer. The subsequent adsorption is achieved through molecular diffusion of the dye molecules into pores of magnetite NPs.^54^

**Table 3:** Average adsorption capacities *q*_*t*_ of EB adsorption on magnetite samples. Standard deviation (STD) of three replicates is mentioned

Sample	*t* (min)	*q*_*t*_ (mg g^−1^)	STD	*t* (min)	*q*_*t*_ (mg g^−1^)	STD	*t* (min)	*q*_*t*_ (mg g^−1^)	STD	*t* (min)	*q*_*t*_ (mg g^−1^)	STD
EB/ARM–Fe_3_O_4_	05	09.15	0.73	10	13.54	0.64	15	17.29	0.71	20	20.55	0.59
EB/ROS–Fe_3_O_4_	05	08.03	0.64	10	11.92	0.59	15	15.06	0.69	20	17.69	0.72
EB/MAT–Fe_3_O_4_	05	04.62	0.75	10	08.24	0.72	15	11.03	0.73	20	13.90	0.78
EB/JUN–Fe_3_O_4_	05	04.05	0.74	10	06.86	0.59	15	09.40	0.62	20	11.83	0.78
EB/ARM–Fe_3_O_4_	25	22.82	0.81	30	25.22	0.59	35	25.29	0.65	40	25.33	0.52
EB/ROS–Fe_3_O_4_	25	20.84	0.63	30	22.75	0.57	35	22.80	0.52	40	22.84	0.72
EB/MAT–Fe_3_O_4_	25	16.25	0.64	30	17.87	0.78	35	17.95	0.67	40	17.99	0.56
EB/JUN–Fe_3_O_4_	25	13.87	0.68	30	16.71	0.65	35	16.76	0.72	40	16.78	0.71
EB/ARM–Fe_3_O_4_	45	25.35	0.66	50	25.37	0.74	60	25.39	0.78			
EB/ROS–Fe_3_O_4_	45	22.87	0.52	50	22.90	0.53	60	22.92	0.71			
EB/MAT–Fe_3_O_4_	45	18.00	0.81	50	18.01	0.61	60	18.03	0.63			
EB/JUN–Fe_3_O_4_	45	16.79	0.80	50	16.81	0.71	60	16.83	0.74			

**Table 4:** Average adsorption capacities *q*_*t*_ of MO adsorption on magnetite samples. Standard deviation (STD) of three replicates is mentioned

Sample	*t* (min)	*q*_*t*_ (mg g^−1^)	STD	*t* (min)	*q*_*t*_ (mg g^−1^)	STD	*t* (min)	*q*_*t*_ (mg g^−1^)	STD	*t* (min)	*q*_*t*_ (mg g^−1^)	STD
MO/ARM–Fe_3_O_4_	05	04.78	0.65	10	08.44	0.65	15	12.88	0.55	20	15.93	0.66
MO/ROS–Fe_3_O_4_	05	04.42	0.89	10	06.99	0.55	15	11.39	0.59	20	14.45	0.50
MO/MAT–Fe_3_O_4_	05	03.80	0.61	10	05.67	0.63	15	09.07	0.54	20	12.06	0.57
MO/JUN–Fe_3_O_4_	05	02.90	0.59	10	04.57	0.52	15	06.84	0.52	20	08.87	0.68
MO/ARM–Fe_3_O_4_	25	19.70	0.69	30	20.70	0.54	35	20.73	0.70	40	20.75	0.69
MO/ROS–Fe_3_O_4_	25	18.32	0.52	30	19.70	0.61	35	19.73	0.58	40	19.75	0.54
MO/MAT–Fe_3_O_4_	25	13.54	0.69	30	14.70	0.55	35	14.71	0.53	40	14.73	0.58
MO/JUN–Fe_3_O_4_	25	10.89	0.52	30	11.66	0.51	35	11.68	0.78	40	11.70	0.52
MO/ARM–Fe_3_O_4_	45	20.79	0.83	50	20.81	0.74	60	20.82	0.53			
MO/ROS–Fe_3_O_4_	45	19.78	0.85	50	19.79	0.74	60	19.80	0.50			
MO/MAT–Fe_3_O_4_	45	14.74	0.64	50	14.76	0.54	60	14.78	0.46			
MO/JUN–Fe_3_O_4_	45	11.71	0.58	50	11.72	0.56	60	11.73	0.49			

#### 3.5.2 Adsorption experiment kinetics analysis

##### 3.5.2.1 Linear and non-linear pseudo-first-order and pseudo-second-order kinetics

**Fig. 8:**
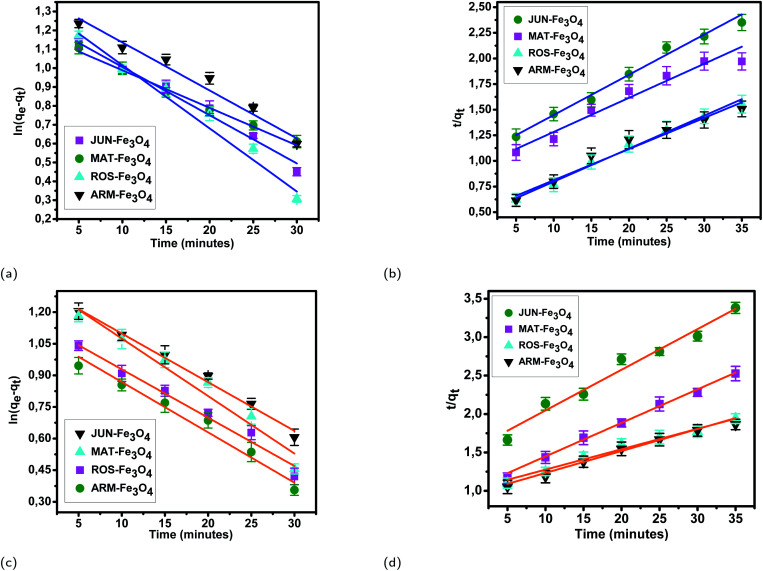
Linear pseudo-first-order, (a) and linear pseudo-second-order (b) kinetic plots of EB adsorption. Linear pseudo-first-order (c), and linear pseudo-second-order (d) kinetic plots of MO adsorption on JUN–Fe_3_O_4_, MAT–Fe_3_O_4_, ROS–Fe_3_O_4_, and ARM–Fe_3_O_4_ surfaces. Error bars represent the standard deviation of three replicates.

**Fig. 9:**
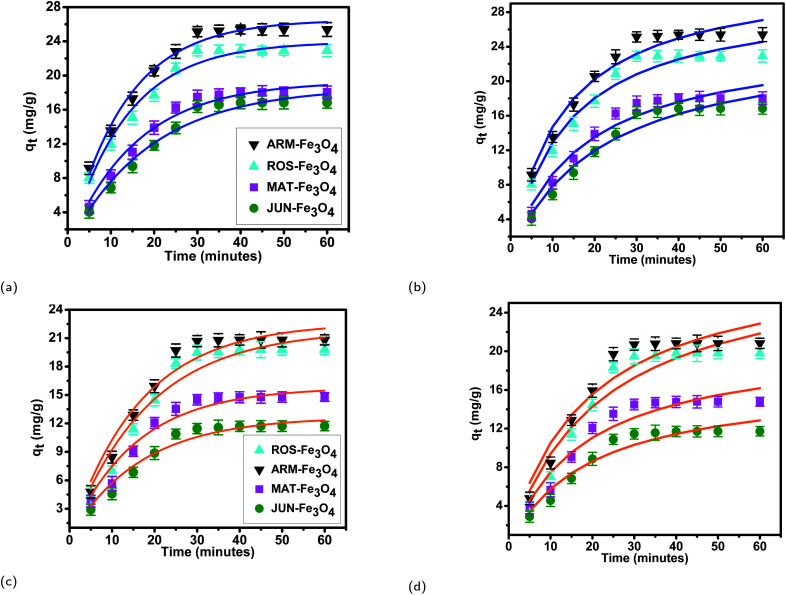
Non-linear pseudo-first-order, (a) and non-linear pseudo-second-order (b) kinetic plots of EB adsorption. Non-linear pseudo-first-order (c), and non-linear pseudo-second-order (d) kinetic plots of MO adsorption on JUN–Fe_3_O_4_, MAT–Fe_3_O_4_, ROS–Fe_3_O_4_, and ARM–Fe_3_O_4_ surfaces. Error bars represent the standard deviation of three replicates.

The results of linear and non-linear pseudo-first-order (PFO) and pseudo-second-order (PSO) kinetics analysis of MO and EB adsorption processes on the four magnetite surfaces (Tables 5 and 6, and Fig. 8 and 9) indicate a good linearity and a good fit of the experimental data to linear pseudo-first-order. However, the comparison with the pseudo-second-order model indicates a poor linearity and a poor fit to the experimental data of this model. Indeed, the equilibrium adsorption capacities *q*_e,cal_ computed from linear pseudo-first-order kinetics plots are in very close agreement with the empirical *q*_e,exp_, unlike *q*_e,cal_ calculated from linear pseudo-second-order plots which are far from empirical *q*_e,exp_.

**Table 5:** Calculated adsorption linear kinetic parameters for the adsorption of MO and EB on the four magnetite surfaces

Sample	*q*_e,exp_ (mg g^−1^)	*q*_e,cal_ (mg g^−1^)	*K*_1_ (min^−1^)	*R* ^2^	*q*_e,cal_ (mg g^−1^)	*K*_2_ (g mg^−1^ min^−1^)	*R* ^2^
EB/ARM–Fe_3_O_4_	25.39	23.85	0.0008	0.983	33.84	0.0016	0.972
EB/ROS–Fe_3_O_4_	22.92	22.93	0.0011	0.989	31.52	0.0020	0.985
EB/MAT–Fe_3_O_4_	18.03	18.61	0.0009	0.982	30.94	0.0010	0.946
EB/JUN–Fe_3_O_4_	16.83	15.50	0.0007	0.991	26.11	0.0014	0.983
MO/ARM–Fe_3_O_4_	20.82	21.52	0.0008	0.988	32.25	0.0008	0.965
MO/ROS–Fe_3_O_4_	19.80	22.85	0.0009	0.987	30.76	0.0007	0.961
MO/MAT–Fe_3_O_4_	14.78	14.50	0.0007	0.996	22.50	0.0020	0.994
MO/JUN–Fe_3_O_4_	11.73	12.27	0.0008	0.989	18.47	0.0019	0.969

**Table 6:** Calculated adsorption non-linear kinetic parameters for the adsorption of MO and EB on the four magnetite surfaces

Sample	*q*_e,exp_ (mg g^−1^)	*q*_e,cal_ (mg g^−1^)	*K*_1_ (min^−1^)	*R* ^2^	*q*_e,cal_ (mg g^−1^)	*K*_2_ (g mg^−1^ min^−1^)	*R* ^2^
EB/ARM–Fe_3_O_4_	25.39	27.53	0.077	0.973	32.56	0.0026	0.964
EB/ROS–Fe_3_O_4_	22.92	23.96	0.075	0.975	29.97	0.0025	0.955
EB/MAT–Fe_3_O_4_	18.03	19.31	0.063	0.975	25.14	0.0023	0.952
EB/JUN–Fe_3_O_4_	16.83	18.62	0.052	0.967	25.07	0.0018	0.952
MO/ARM–Fe_3_O_4_	20.82	22.62	0.060	0.948	29.88	0.0018	0.924
MO/ROS–Fe_3_O_4_	19.80	23.89	0.055	0.925	29.69	0.0015	0.923
MO/MAT–Fe_3_O_4_	14.78	15.79	0.064	0.952	21.05	0.0026	0.926
MO/JUN–Fe_3_O_4_	11.73	12.88	0.060	0.948	17.02	0.0030	0.931

##### 3.5.2.2 Intra-particle diffusion mechanism analysis

**Fig. 10:**
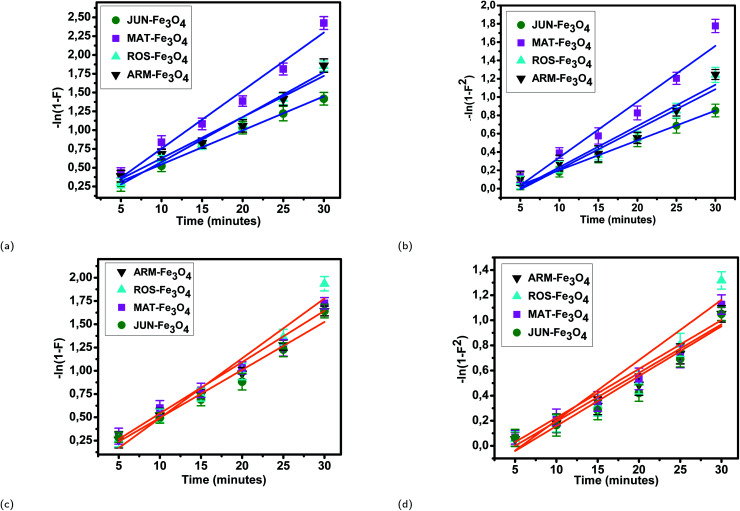
Homogeneous particle diffusion plots of EB adsorption: (a) −ln(1 − *F*) and (b) −ln(1 − *F*^2^). Homogeneous particle diffusion plots of MO adsorption: (c) −ln(1 − *F*) and (d) −ln(1 − *F*^2^), on magnetite surfaces. Error bars represent the standard deviation of three replicates.

The linearity tests of Boyd plots (−ln(1 − *F*) and −ln(1 − *F*^2^)) *versus* time are presented in Fig. 10. The kinetic data correlate well with the homogeneous particle diffusion model as confirmed by the high *R*^2^ values. The results of linear regression analysis for eqn (10) and (12) are presented in Table 7. Film diffusion coefficients *D*_f_ are found to be in the order of 10^−11^ m^2^ s^−1^, while intra-particle diffusion coefficients *D*_p_ are found to be in the order of 10^−20^ m^2^ s^−1^. Michelson *et al.*^55^ reported that the adsorption mechanism is controlled by film diffusion and that film diffusion is in control at *D*_f_ ranging from 10^−10^ to 10^−12^ m^2^ s^−1^, while intra-particle diffusion is the rate-limiting step at *D*_p_ in the range of 10^−15^ to 10^−18^ m^2^ s^−1^. Indeed, the obtained results indicate that film diffusion is the step that controls the adsorption mechanism of MO and EB on magnetite surfaces, which is in agreement with the pseudo-first-order kinetic model.

**Table 7:** Calculated homogeneous particle diffusion parameters of MO and EB on ARM–Fe_3_O_4_, ROS–Fe_3_O_4_, MAT–Fe_3_O_4_ and JUN–Fe_3_O_4_ samples

Sample	*r*_0_ × 10^−9^ (m)	*k*_p_ × 10^−3^ (1/*s*)	*R* ^2^	*D*_p_ × 10^−19^ (m^2^ s^−1^)	*k*_f_ × 10^−3^ (1/*s*)	*R* ^2^	*D*_f_ × 10^−12^ (m^2^ s^−1^)
EB/ARM–Fe_3_O_4_	41.94	2.18	0.963	3.89	1.44	0.953	03.38
EB/ROS–Fe_3_O_4_	39.89	2.26	0.985	3.68	1.51	0.962	05.91
EB/MAT–Fe_3_O_4_	33.13	2.51	0.977	2.80	2.08	0.956	14.97
EB/JUN–Fe_3_O_4_	29.27	1.89	0.993	1.65	1.08	0.998	08.13
MO/ARM–Fe_3_O_4_	41.94	1.79	0.990	3.67	1.30	0.965	07.67
MO/ROS–Fe_3_O_4_	39.89	2.12	0.963	3.43	1.60	0.930	10.62
MO/MAT–Fe_3_O_4_	33.13	1.77	0.977	1.98	1.32	0.951	14.28
MO/JUN–Fe_3_O_4_	29.27	1.75	0.970	1.46	1.28	0.970	18.97

#### 3.5.3 Activation thermodynamic parameters of MO and EB adsorption on magnetite surfaces

**Fig. 11:**
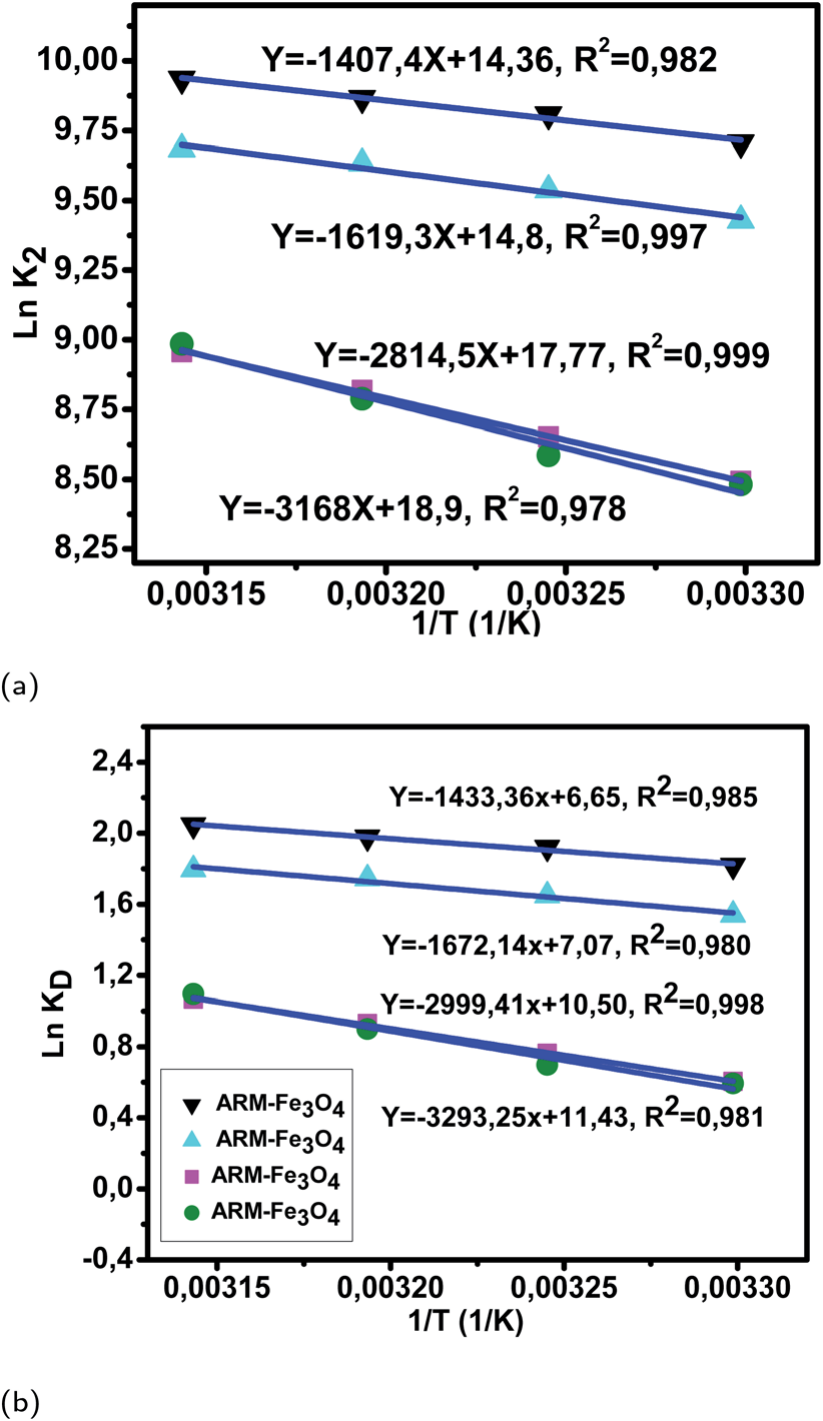
(a) Plots of ln *K*_2_*versus* 1/*T* of EB adsorption on Fe_3_O_4_ surfaces. (b) Plots of ln *K*_D_*versus* 1/*T* of EB adsorption on Fe_3_O_4_ surfaces.

**Fig. 12:**
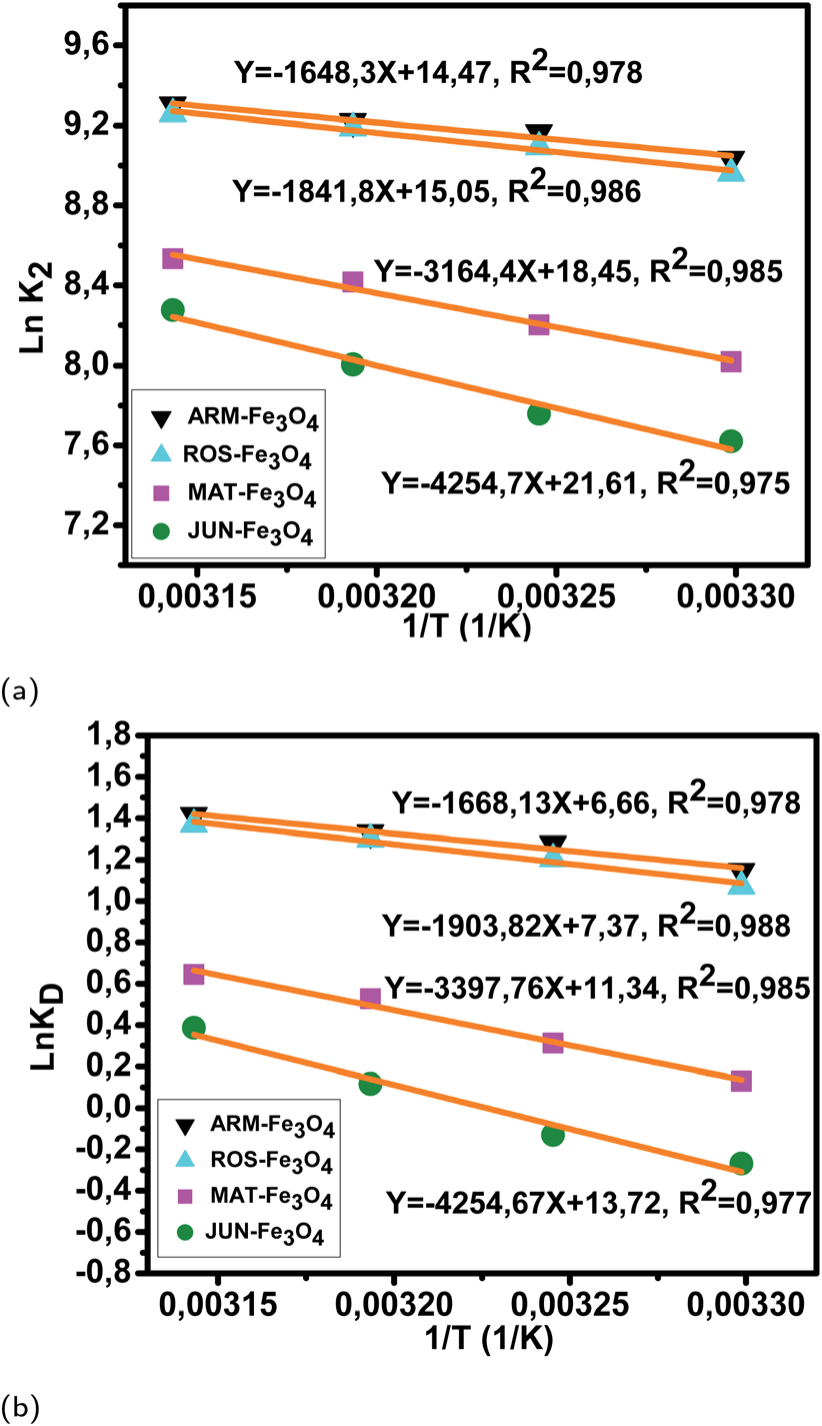
(a) Plots of ln *K*_2_*versus* 1/*T* of MO adsorption on Fe_3_O_4_ surfaces. (b) Plots of ln *K*_D_*versus* 1/*T* of MO adsorption on Fe_3_O_4_ surfaces.

The calculated activation enthalpy Δ*H*^0^, entropy Δ*S*^0^, and free energy Δ*G*^0^ are listed in Tables 8 and 9. Δ*H*^0^ and Δ*S*^0^ of EB/plant–Fe_3_O_4_ systems were respectively calculated from the slopes and intercepts of the Arrhenius linear plots ln *K*_D_*versus* 1/*T* (Fig. 11b). Likewise, Δ*H*^0^ and Δ*S*^0^ of MO/plant–Fe_3_O_4_ systems were respectively calculated from the slopes and intercepts of the Arrhenius linear plots ln *K*_D_*versus* 1/*T* (Fig. 12b). Activation enthalpy in all EB/plant–Fe_3_O_4_ and MO/plant–Fe_3_O_4_ systems are positive, which indicates the endothermic nature of the adsorption processes and possible strong bonding between dye molecules and functional hydroxyl groups on Fe_3_O_4_ surfaces. The found activation enthalpies of the EB/JUN–Fe_3_O_4_ (6.54 kcal mol^−1^) and MO/JUN–Fe_3_O_4_ (8.67 kcal mol^−1^) systems are the highest ones, and those of EB/ARM–Fe_3_O_4_ (2.85 kcal mol^−1^) and MO/ARM–Fe_3_O_4_ (3.31 kcal mol^−1^) systems are the lowest ones. This indicates that the bonds between EB and MO molecules and active site hydroxyl groups on the JUN–Fe_3_O_4_ surface are the strongest ones, followed by those on MAT–Fe_3_O_4_, then on ROS–Fe_3_O_4_, and finally on ARM–Fe_3_O_4_ surfaces.

**Table 8:** Calculated thermodynamic parameters for EB adsorption on the four plant–Fe_3_O_4_ surfaces

Sample	*T* (K)	ln *K*_D_	ln *K*_2_	*E*_a_ (kcal mol^−1^)	Δ*H*^0^ (kcal mol^−1^)	Δ*S*^0^ (cal mol^−1^ K^−1^)	Δ*G*^0^ (kcal mol^−1^)
EB/ARM–Fe_3_O_4_	303.15	1.82	9.71	2.79	2.85	13.03	−1.09
	308.15	1.92	9.81				−1.18
	313.15	1.98	9.84				−1.23
	318.15	2.05	9.94				−1.29
EB/ROS–Fe_3_O_4_	303.15	1.54	9.43	3.21	3.32	14.04	−0.93
	308.15	1.65	9.54				−1.00
	313.15	1.75	9.61				−1.09
	318.15	1.80	9.68				−1.14
EB/MAT–Fe_3_O_4_	303.15	0.61	8.49	5.59	5.96	20.86	−0.36
	308.15	0.76	8.65				−0.47
	313.15	0.93	8.73				−0.58
	318.15	1.07	8.94				−0.67
EB/JUN–Fe_3_O_4_	303.15	0.59	8.48	6.29	6.54	22.70	−0.35
	308.15	0.70	8.59				−0.43
	313.15	0.90	8.71				−0.56
	318.15	1.07	8.95				−0.66

**Table 9:** Calculated thermodynamic parameters for MO adsorption on the four plant–Fe_3_O_4_ surfaces

Sample	*T* (K)	ln *K*_D_	ln *K*_2_	*E*_a_ (kcal mol^−1^)	Δ*H*^0^ (kcal mol^−1^)	Δ*S*^0^ (cal mol^−1^ K^−1^)	Δ*G*^0^ (kcal mol^−1^)
MO/ARM–Fe_3_O_4_	303.15	1.14	9.03	3.27	3.31	13.24	−0.69
	308.15	1.28	9.17				−0.78
	313.15	1.33	9.21				−0.83
	318.15	1.41	9.30				−0.89
MO/ROS–Fe_3_O_4_	303.15	1.07	8.96	3.66	3.78	14.63	−0.65
	308.15	1.20	9.09				−0.74
	313.15	1.30	9.16				−0.81
	318.15	1.37	9.26				−0.87
MO/MAT–Fe_3_O_4_	303.15	0.13	8.02	6.28	6.75	22.54	−0.078
	308.15	0.31	8.20				−0.19
	313.15	0.53	8.30				−0.33
	318.15	0.64	8.53				−0.41
MO/JUN–Fe_3_O_4_	303.15	−0.27	7.62	8.45	8.67	27.27	+0.16
	308.15	−0.13	7.76				+0.08
	313.15	+0.12	8.00				−0.07
	318.15	+0.39	8.28				−0.24

The activation entropies in all EB/plant–Fe_3_O_4_ and MO/plant–Fe_3_O_4_ systems are positive, which reveals the affinity of Fe_3_O_4_ surfaces for EB and MO molecules. The increasing randomness at the EB/plant–magnetite and MO/plant–magnetite solution interfaces indicates that significant changes in the number of surface active hydroxyl groups occurred in the internal structure of Fe_3_O_4_ surfaces. However, activation entropies of EB/JUN–Fe_3_O_4_ (22.70 cal mol^−1^ K^−1^) and MO/JUN–Fe_3_O_4_ (27.27 cal mol^−1^ K^−1^) systems are the highest ones, and those of EB/ARM–Fe_3_O_4_ (13.03 cal mol^−1^ K^−1^) and MO/ARM–Fe_3_O_4_ (13.24 cal mol^−1^ K^−1^) systems are the lowest ones. This indicates that the changes occurring in the structure of the JUN–Fe_3_O_4_ surface are the greatest ones, followed by those of MAT–Fe_3_O_4_, then ROS–Fe_3_O_4_, and finally ARM–Fe_3_O_4_ surfaces.^56,57^

The activation free energies of EB/ARM–Fe_3_O_4_ (−1.09, −1.17, −1.23, and −1.29 kcal mol^−1^), EB/ROS–Fe_3_O_4_ (−0.93, −1.00, −1.09, and −1.14 kcal mol^−1^), EB/MAT–Fe_3_O_4_ (−0.36, −0.47, −0.58, and −0.67 kcal mol^−1^), and EB/JUN–Fe_3_O_4_ (−0.35, −0.43, −0.56, and −0.66 kcal mol^−1^) systems are negative. However, activation energies of the EB/ARM–Fe_3_O_4_ system are more negative than those of EB/ROS–Fe_3_O_4_, EB/MAT–Fe_3_O_4_ and EB/JUN–Fe_3_O_4_ systems, which indicates the feasibility of the EB adsorption process and its spontaneous nature with more EB adsorption on ARM–Fe_3_O_4_, then on ROS–Fe_3_O_4_, next on MAT–Fe_3_O_4_, and finally on JUN–Fe_3_O_4_ surfaces.

The activation free energies of MO/ARM–Fe_3_O_4_ (−0.69, −0.78, −0.83, and −0.89 kcal mol^−1^), MO/ROS–Fe_3_O_4_ (−0.65, −0.74, −0.81, and −0.87 kcal mol^−1^), and MO/MAT–Fe_3_O_4_ (−0.078, −0.19, −0.33, and −0.41 kcal mol^−1^) systems are negative. However, activation energies of the MO/ARM–Fe_3_O_4_ system are more negative than those of MO/ROS–Fe_3_O_4_ and MO/MAT–Fe_3_O_4_ systems, which indicates the feasibility of the MO adsorption process and its spontaneous nature with more MO adsorption on ARM–Fe_3_O_4_ than on ROS–Fe_3_O_4_ surfaces. In the MO/JUN–Fe_3_O_4_ system, the values of activation free energy are negative only at 313.15 K and 318.15 K (−0.072 and −0.24 kcal mol^−1^, respectively), while positive values are found at 303.15 K and 308.15 K (0.16 and 0.079 kcal mol^−1^, respectively) revealing that activated MO/Fe_3_O_4_ complexes are in an excited form in the transition state.^56^ This leads to the spontaneity of MO adsorption at 313.15 K and 318.15 K.

As presented in Table 8, the found activation energies (*E*_a_) for EB adsorption on ARM–Fe_3_O_4_, ROS–Fe_3_O_4_, MAT–Fe_3_O_4_, and JUN–Fe_3_O_4_ surfaces are respectively: 2.79, 3.21, 5.59, and 6.29 kcal mol^−1^. *E*_a_ is calculated from the slopes of the Arrhenius linear plots ln *K*_2_*versus* 1/*T* (Fig. 11a). As presented in Table 9, the found activation energies (*E*_a_) for MO adsorption on ARM–Fe_3_O_4_, ROS–Fe_3_O_4_, MAT–Fe_3_O_4_, and JUN–Fe_3_O_4_ surfaces are respectively: 3.27, 3.66, 6.28, and 8.45 kcal mol^−1^. *E*_a_ is calculated from the slopes of Arrhenius linear plots ln *K*_2_*versus* 1/*T* (Fig. 12a). The found low *E*_a_ suggests that EB and MO adsorption processes on all plant–Fe_3_O_4_ surfaces proceeded with low energy barriers and can be achieved at relatively low temperatures. As it is known that the activation energy *E*_a_ of physical adsorption ranges from 1.2 to 12 kcal mol^−1^, and from 14.3 to 191 kcal mol^−1^ for chemical adsorption,^58^ the adsorption processes of EB and MO on all plant–Fe_3_O_4_ are physical in nature.

#### 3.5.4 Effect of temperature on EB and MO adsorption yields and capacities on magnetite surfaces

**Fig. 13:**
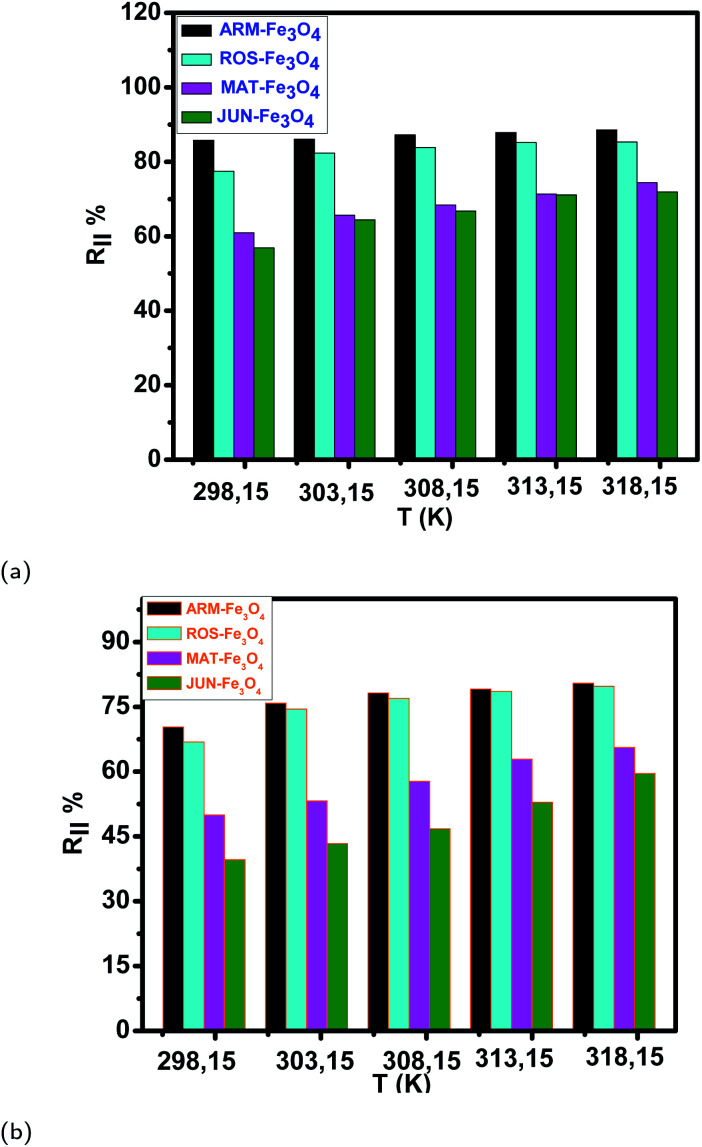
(a and b) Temperature effect on EB and MO adsorption yields on plant–Fe_3_O_4_ surfaces in the range of 303.15–318.15 K over 20 minutes, respectively.

The thermodynamic studies of EB and MO adsorption on all four magnetite samples are evaluated by assessing the efficiency of degradation of EB and MO by increasing the temperature from 303.15 K to 318.15 K over 20 minutes. Fig. 13a and b, and Table 10 present the comparison of EB and MO adsorption yields and capacities on the four Fe_3_O_4_ surfaces at ambient temperature and in the temperature range of 303.15–318.15 K. The data show that EB and MO adsorption yields and capacities increase with the increase of temperature in all adsorption experiments, which confirms the endothermic nature of the adsorption processes as discussed in Section 3.5.3.

**Table 10:** Temperature effect on EB and MO adsorption yields and capacities on the four plant–Fe_3_O_4_ surfaces

Sample	298.15 K		303.15 K		308.15 K		313.15 K		318.15 K	
	*q*_e_ (mg g^−1^)	*R* (%)	*q*_e*T*_ (mg g^−1^)	*R*_*T*_ (%)	*q*_e*T*_ (mg g^−1^)	*R*_*T*_ (%)	*q*_e*T*_ (mg g^−1^)	*R*_*T*_ (%)	*q*_e*T*_ (mg g^−1^)	*R*_*T*_ (%)
EB/ARM–Fe_3_O_4_	25.39	86.05	25.47	86.24	25.91	87.21	26.00	87.84	26.21	88.56
EB/ROS–Fe_3_O_4_	22.92	77.44	24.37	82.34	24.83	83.87	25.2	85.14	25.09	85.77
EB/MAT–Fe_3_O_4_	18.03	60.91	19.44	65.66	20.24	68.38	21.12	71.35	22.19	74.95
EB/JUN–Fe_3_O_4_	16.83	56.88	19.07	64.41	19.76	66.76	21.04	71.08	21.52	72.70
MO/ARM–Fe_3_O_4_	20.82	70.32	21.92	74.05	22.29	75.32	22.43	75.76	22.58	76.31
MO/ROS–Fe_3_O_4_	19.80	66.88	20.59	69.55	20.93	70.72	21.65	73.15	22.05	74.50
MO/MAT–Fe_3_O_4_	14.78	49.94	15.76	53.24	17.09	57.75	18.61	62.88	19.41	65.59
MO/JUN–Fe_3_O_4_	11.72	39.61	12.82	43.33	13.84	46.76	15.65	52.88	17.63	59.55

The tendency of adsorption capacities and yields on the four magnetite surfaces is the same in EB and MO adsorption processes at 298.15 K and after increasing the temperature from 303.15 to 318.15 K. In EB and MO adsorption processes (298.15 K), the highest adsorption capacities were on ARM–Fe_3_O_4_, then on ROS–Fe_3_O_4_, next on MAT–Fe_3_O_4_, and finally on JUN–Fe_3_O_4_ NPs. After the exposure of EB/plant–magnetite and MO/plant–magnetite systems to heat in the temperature range of 303.15–318.15 K for 20 minutes, the order of adsorption capacities was the same.

### 3.6 Influence of the mediating plant extract's acidity on preferential attachment of chromophore and auxochrome groups of MO and EB on magnetite NPs

To study the adsorption of MO and EB on these four magnetite samples, all adsorption experiments were conducted under the same conditions including solution pH = 4 so as to eliminate solution pH effect on the adsorption.^7,14,19^

**Fig. 14:**
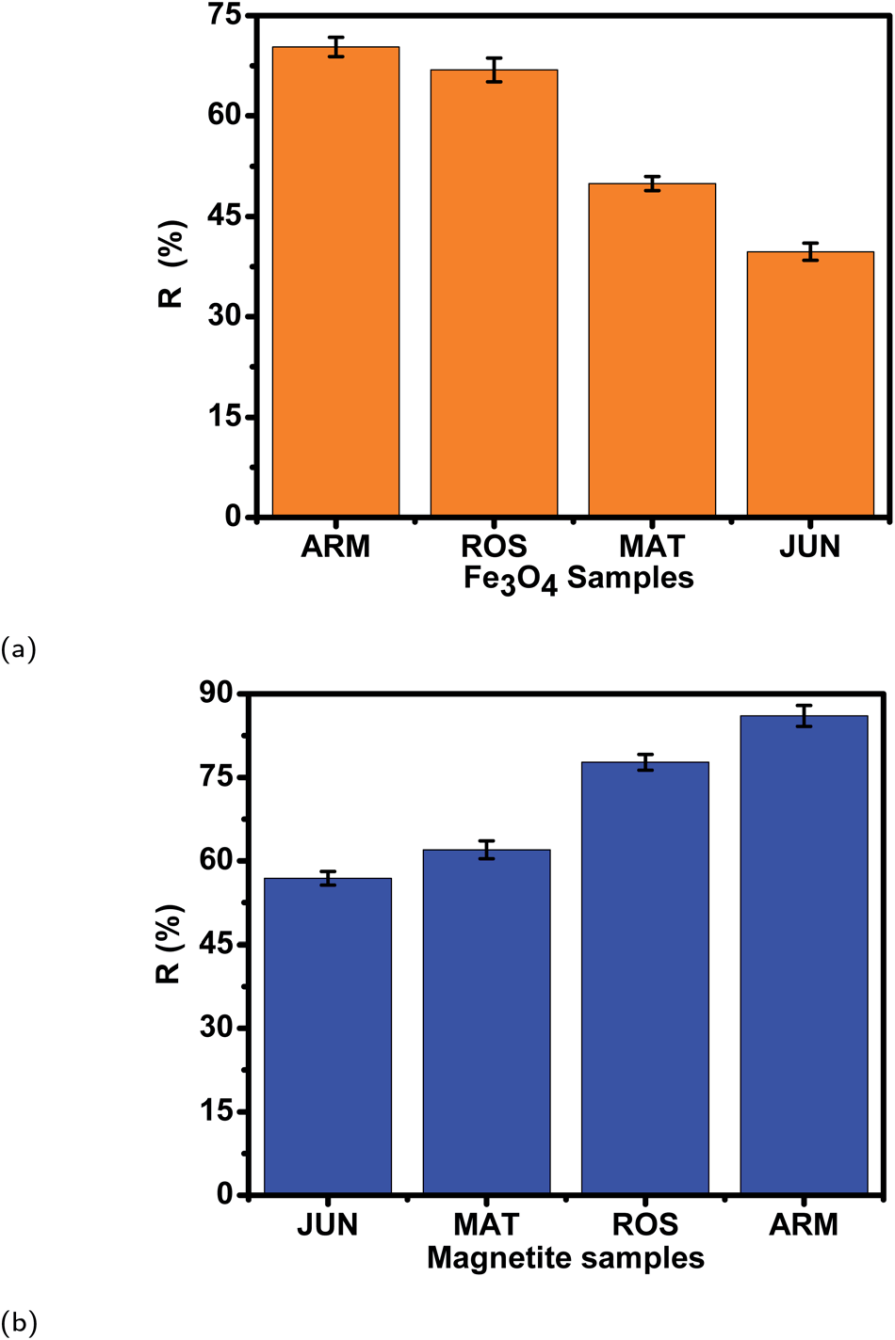
Adsorption yields of (a) MO and (b) EB dyes on different magnetite surfaces. Error bars represent the standard deviation of three replicates.

Table 11 and Fig. 14 show that the adsorption yields and capacities of dyes differed on the four Fe_3_O_4_ NPs according to the pH of plant extracts used in magnetite sample synthesis. EB and MO anions were highly adsorbed on the ARM–Fe_3_O_4_ surface with achieved adsorption yields and capacities of 86.05%, 25.39 mg g^−1^ and 70.31%, 20.82 mg g^−1^, respectively, then on the ROS–Fe_3_O_4_ surface with achieved adsorption yields of 77.71%, 22.92 mg g^−1^ and 66.88%, 19.80 mg g^−1^, respectively, next on the MAT–Fe_3_O_4_ surface with achieved adsorption yields of 61.98%, 18.03 mg g^−1^ and 49.94%, 14.78 mg g^−1^, respectively, and finally, on the JUN–Fe_3_O_4_ surface where the adsorption yields and capacities of EB and MO achieved values of only 56.88%, 16.83 mg g^−1^ and 39.61%, 11.73 mg g^−1^, respectively.When magnetite is immersed in an aqueous acidic solution, it develops its surface charge *via* the protonation and deprotonation of ≡FeOH sites on its surface according to the following equation:^59^

23 ≡FeOH^+^_2_ ↔ ≡FeOH^0^ + *H*^+^_sol_ (p*k*_a1_ = 5.1)

where ≡FeOH^+^_2_ and ≡FeOH^0^ are respectively the protonated positively charged surface group with two dissociable H^+^, and the neutral surface group with one dissociable H^+^. p*K*_a1_ = 5.1 is the intrinsic acidity constant determined by Davis *et al.*^59^ for magnetite. ≡FeOH^+^_2_ known as the Brønsted acid site is an electron-pair acceptor and it is able to transfer *H*^+^ from the solid to the adsorbed molecule, implying an H-bond with the surface. Meanwhile, ≡FeOH^0^ known as the Lewis acid site is an electron-pair acceptor from the adsorbed molecule, implying a coordinated bond with the surface.^59^

**Table 11:** Average adsorption yields and capacities of MO and EB achieved on magnetite surfaces

Adsorbent	*q*_e_ (EB) (mg g^−1^)	*R* (EB) (%)	*q*_e_ (MO) (mg g^−1^)	*R* (MO) (%)	pH of plant extract
ARM–Fe_3_O_4_	25.39	86.05	20.82	70.31	5.25
ROS–Fe_3_O_4_	22.92	77.71	19.80	66.88	5.05
MAT–Fe_3_O_4_	18.03	61.98	14.78	49.94	4.63
JUN–Fe_3_O_4_	16.83	56.88	11.73	39.61	3.69

In order to study how the plant extract's acidity impacted preferential attachment of dyes' chromophore and auxochrome groups on synthesized magnetite surfaces, the free chromophore and auxochrome groups that were not attached to magnetite surfaces have been deeply analyzed in all dyes' residual solutions using FTIR spectroscopy, so as to perceive preferential attachment of chromophore and auxochrome groups on each surface and hence determine Brønsted and Lewis acid site densities. Based on this analysis, it was possible to infer Brønsted and Lewis acid site densities on each magnetite surface. For this purpose, after the accomplishment of the adsorption in all experiments, the solid and liquid fractions were separated using a centrifuge. In the next sections, the analysis of residual dye chemistry changes of MO and EB will be studied in detail by comparison between FTIR spectra of MO and EB reference solution chemistry, and the chemistry of their residual solutions.

In the rest of this paper, MO residual solutions will be denoted as MO/ARM–Fe_3_O_4_, MO/ROS–Fe_3_O_4_, MO/MAT–Fe_3_O_4_, and MO/JUN–Fe_3_O_4_. Meanwhile, EB residual solutions will be denoted as EB/ARM–Fe_3_O_4_, EB/ROS–Fe_3_O_4_, EB/MAT–Fe_3_O_4_, and EB/JUN–Fe_3_O_4_.

#### 3.6.1 Analysis of MO/plant–magnetite residual solution chemistry changes

**Fig. 15:**
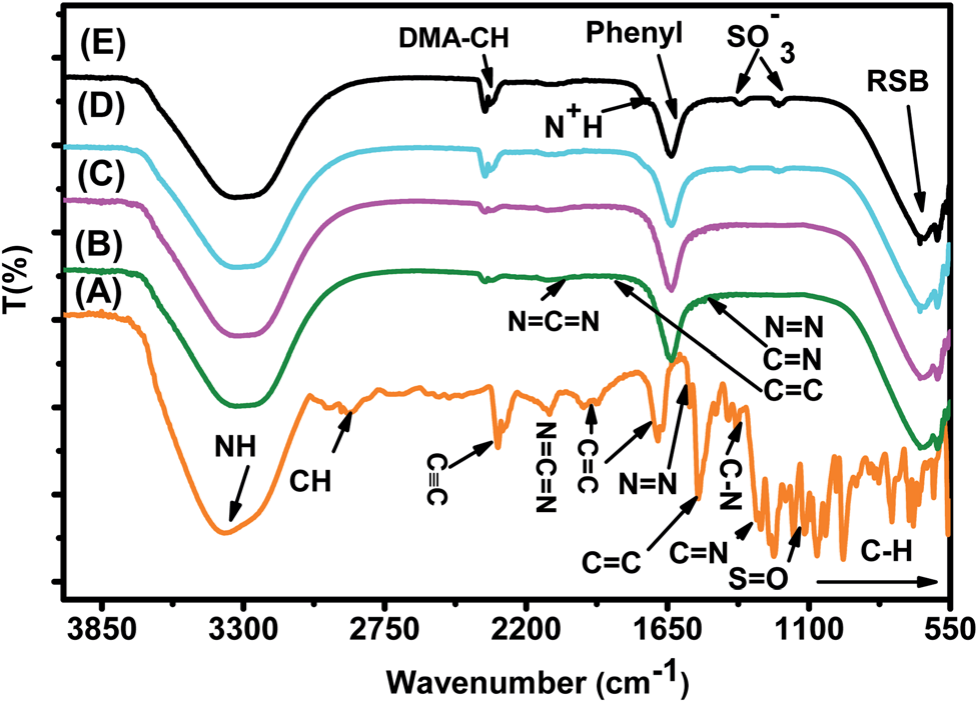
FTIR spectra of (A) the MO reference solution, (B) MO/JUN–Fe_3_O_4_, (C) MO/MAT–Fe_3_O_4_, (D) MO/ROS–Fe_3_O_4_, and (E) MO/ARM–Fe_3_O_4_ residual solutions.

The FTIR spectrum of the MO reference solution (Fig. 15A) shows a broad peak of NH bond stretching of phenyldiazonium groups at 3378.48 cm^−1^, aromatic CH ring stretching at 2915.93 cm^−1^,^60^ C≡C at 2300–2100 cm^−1^, N=C=N at 2100.95 cm^−1^,^61^ aromatic C=C ring stretching at 1655.87 cm^−1^^62^ and at 1522.60 cm^−1^,^63^ N=N at 1569.87 cm^−1^ and at 1416.02 cm^−1^,^62,64^ C–N at 1355.01 cm^−1^ and 1160.08 cm^−1^,^62,64^ C=N at 1304.74 cm^−1^,^64^ S=O at 1112.02 cm^−1^,^63^ and C–H bending vibrations of the aromatic ring at 1047.03 cm^−1^^64^ and at 1000–550 cm^−1^.^65^

In Fig. 15, spectra (B)–(E) present the FTIR spectra of MO/JUN–Fe_3_O_4_, MO/MAT–Fe_3_O_4_, MO/ROS–Fe_3_O_4_, and MO/ARM–Fe_3_O_4_ residual solutions, respectively. The disappearance of certain peaks and the appearance of new ones indicate that the MO/plant–magnetite residual solution chemistry was changed as a result of MO adsorption on magnetite surfaces. The newly appeared peaks correspond to SO^−^_3_, 
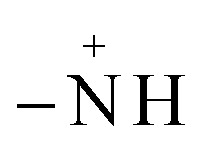
, asymmetric CH, phenyl, and the ring skeleton of benzene groups (RSB). More details about the identification of these peaks in MO/plant–magnetite residual solutions will be given in the next paragraphs, for the purpose of investigating the preferential attachment of chromophore and auxochrome groups on all four magnetite surfaces.

The decomposition of the dye is significantly accelerated by the presence of acidic centers at the surface.^66^

##### 3.6.1.1 Analysis of preferential attachment of chromophore groups in the adsorption of MO

New peaks of the NH bond of phenyldiazonium groups shifted from 3378.48 cm^−1^ to around 3308 cm^−1^ in all four residual solutions (Fig. 15), with however different peak areas. Furthermore, all spectra in Fig. 15 show that new peaks, with however different areas, appear at around 1633 cm^−1^. These peaks correspond to the C=C bond of not attached phenyl groups.^67^ Additionally, new peaks appear, with different areas, at around 646 cm^−1^, which correspond to the CH of the ring skeleton of benzene.^21^ The peaks of these groups in the FTIR spectrum of MO/ARM–Fe_3_O_4_ are the narrowest ones, followed by those of MO/ROS–Fe_3_O_4_, then those of MO/MAT–Fe_3_O_4_, and finally, the broadest one is in the FTIR spectrum of MO/JUN–Fe_3_O_4_. This indicates that phenyldiazonium, phenyl, and benzene groups were more attached on ARM–Fe_3_O_4_, next on ROS–Fe_3_O_4_, then on MAT–Fe_3_O_4_, and finally, on JUN–Fe_3_O_4_.

Minor peaks corresponding to N=N, C=C, and C=N groups, linked to the benzene ring, appear in the FTIR spectra of MO/MAT–Fe_3_O_4_ and MO/JUN–Fe_3_O_4_. Those peaks appearing in the FTIR spectrum of MO/JUN–Fe_3_O_4_ are slightly more intense than in that of MO/MAT–Fe_3_O_4_, while they do not appear in the FTIR spectra of MO/ROS–Fe_3_O_4_ and MO/ARM–Fe_3_O_4_ (Fig. 15). The lack of these groups in MO/ROS–Fe_3_O_4_ and MO/ARM–Fe_3_O_4_ indicates their complete attachment on ROS–Fe_3_O_4_ and ARM–Fe_3_O_4_ surfaces. However, these groups were almost completely attached on MAT–Fe_3_O_4_ and they were less attached on the JUN–Fe_3_O_4_ surface.

**Fig. 16:**
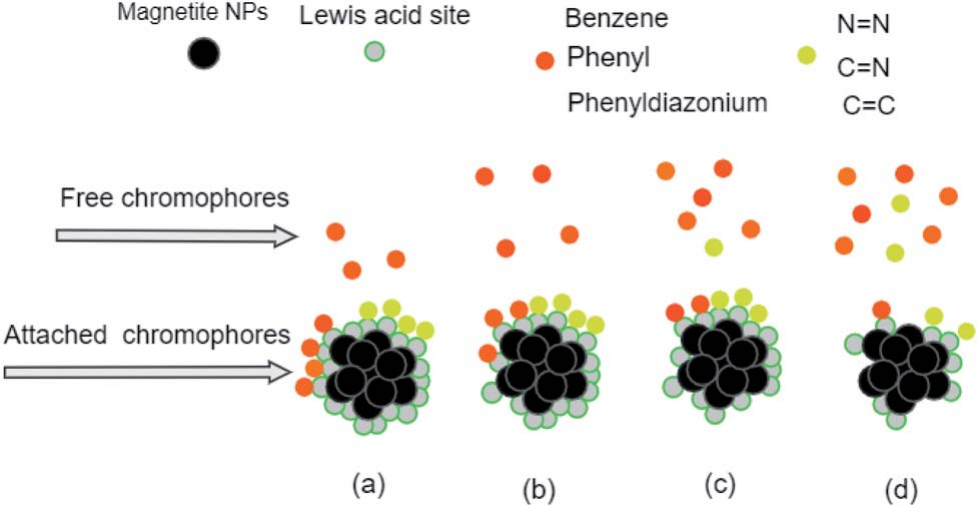
Preferential attachment of MO chromophore groups on (a) ARM–Fe_3_O_4_, (b) ROS–Fe_3_O_4_, (c) MAT–Fe_3_O_4_, and (d) JUN–Fe_3_O_4_ surfaces. The chromophore groups are grouped into two categories according to their behaviour in the attachment on the magnetite surface.

The analysis of preferential attachment of chromophore groups shows, as summarized in Fig. 16, that:

• On JUN–Fe_3_O_4_, all MO chromophore groups were less attached compared to on the three other magnetite NPs.

• On MAT–Fe_3_O_4_, N=N, C=C, and C=N groups were almost completely attached while other groups were more attached compared to on JUN–Fe_3_O_4_ and less attached compared to on ARM–Fe_3_O_4_ and ROS–Fe_3_O_4_.

• On ROS–Fe_3_O_4_, N=N, C=C, and C=N were completely attached, while other groups were less attached than on ARM–Fe_3_O_4_ and more attached than on MAT–Fe_3_O_4_ and JUN–Fe_3_O_4_.

• On ARM–Fe_3_O_4_, N=N, C=C, and C=N were completely attached, whereas phenyldiazonium, phenyl, and benzene groups were more attached on ARM–Fe_3_O_4_ than on the other magnetite NPs.

As it is known that chromophore groups prefer to attach to Lewis acid sites, it is possible to infer that the density of Lewis acid sites of ARM–Fe_3_O_4_ is the highest one, followed by that of ROS–Fe_3_O_4_, then that of MAT–Fe_3_O_4_, and finally, that of JUN–Fe_3_O_4_.

The functional group attachment analysis results are consistent with MO adsorption yields, being the highest on ARM–Fe_3_O_4_ (70.31%), then on ROS–Fe_3_O_4_ (66.98%), next on MAT–Fe_3_O_4_ (49.94%), and finally on JUN–Fe_3_O_4_ (39.61%). This is due to the fact that most of the MO functional groups are chromophores.

##### 3.6.1.2 Analysis of preferential attachment of auxochrome groups in the adsorption of MO

As illustrated in Fig. 15, FTIR spectra of MO/ARM–Fe_3_O_4_ and MO/ROS–Fe_3_O_4_ show new peaks at 1367.14 and 1202.44 cm^−1^, and at 1365.24 and 1203.61 cm^−1^, respectively. These peaks correspond to SO^−^_3_ sulfonic acid groups.^62^ It is remarked that these peaks are slightly more intense in the FTIR spectrum of MO/ARM–Fe_3_O_4_ than in that of MO/ROS–Fe_3_O_4_. However, these peaks do not appear in the spectra of MO/MAT–Fe_3_O_4_ and MO/JUN–Fe_3_O_4_. This indicates that SO^−^_3_ groups were completely attached on MAT–Fe_3_O_4_ and JUN–Fe_3_O_4_ surfaces, whereas they were more attached on ROS–Fe_3_O_4_ compared to the ARM–Fe_3_O_4_ surface.

Furthermore, FTIR spectra of all four residual solutions (Fig. 15) show that new peaks of asymmetric vibration of CH of CH_3_ in ionized dimethylamine (DMA) 
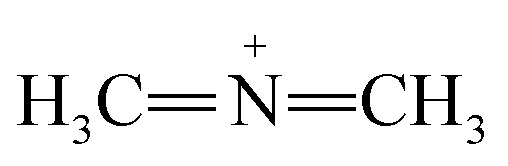
 groups appear at around 2342 cm^−1^,^68^ and their N=C=N and 
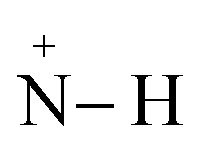
 stretching bonds at around 2090 cm^−1^ and 1739.94 cm^−1^, respectively, with however remarkably different areas. The broadest peak area appears in the FTIR spectrum of MO/ARM–Fe_3_O_4_, next in that of MO/ROS–Fe_3_O_4_, then in that of MO/MAT–Fe_3_O_4_, and finally, the narrowest ones are in the FTIR spectrum of MO/JUN–Fe_3_O_4_. This leads to the conclusion that 
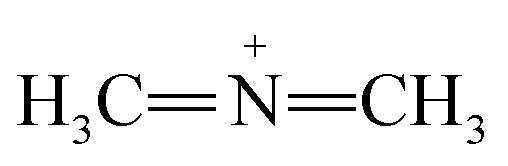
 groups were more attached on JUN–Fe_3_O_4_, then on MAT–Fe_3_O_4_, next on ROS–Fe_3_O_4_, and finally, on ARM–Fe_3_O_4_.

The analysis of preferential attachment of auxochrome groups shows that:

• On ARM–Fe_3_O_4_, all MO auxochrome groups were less attached compared to on the three other magnetite NPs.

• On ROS–Fe_3_O_4_, sulphonic acid and dimethylamine groups were more attached compared to on ARM–Fe_3_O_4_.

• On MAT–Fe_3_O_4_, sulphonic acid groups were completely attached; however, dimethylamine groups were less attached compared to on JUN–Fe_3_O_4_.

• On JUN–Fe_3_O_4_, sulphonic acid groups were completely attached, and dimethylamine groups were more attached compared to on MAT–Fe_3_O_4_.

This leads to the conclusion that the density of Brønsted acid sites of JUN–Fe_3_O_4_ is the highest one, followed by that of MAT–Fe_3_O_4_, then that of ROS–Fe_3_O_4_, and finally, that of ARM–Fe_3_O_4_.

#### 3.6.2 Analysis of EB/plant–magnetite residual solution chemistry changes

**Fig. 17:**
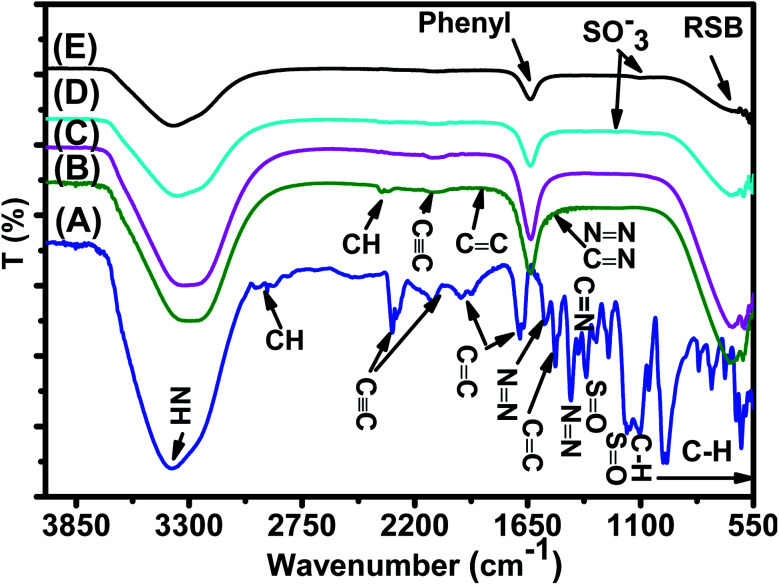
FTIR spectra of (A) the EB reference solution, (B) EB/JUN–Fe_3_O_4_, (C) EB/MAT–Fe_3_O_4_, (D) EB/ROS–Fe_3_O_4_, and (E) EB/ARM–Fe_3_O_4_ residual solutions.

The FTIR spectrum of the EB reference solution presented in Fig. 17 shows a broad peak of the NH bond of the phenyldiazonium ring at 3375.89 cm^−1^,^60^ aromatic CH ring stretching at 2920.97 cm^−1^,^60^ C≡C at 2300–2100 cm^−1^,^61^ aromatic C=C ring stretching at 1943.10 cm^−1^, 1658.11 cm^−1^,^62^ and 1519.25 cm^−1^,^63^ N=N bond at 1569.75 cm^−1^ and 1418.76 cm^−1^,^62,64^ C–N at 1351.98 cm^−1^ and 1164.01 cm^−1^,^62,64^ C=N at 1306.03 cm^−1^,^64^ S=O at 1114.67 cm^−1^,^63,64^ and C–H bending vibration of the aromatic ring at 1042.40 cm^−1^^64^ and 1000–550 cm^−1^.^65^

FTIR spectra of EB/JUN–Fe_3_O_4_, EB/MAT–Fe_3_O_4_, EB/ROS–Fe_3_O_4_, and EB/ARM–Fe_3_O_4_ are presented in Fig. 17. The disappearance of peaks and the appearance of new peaks indicate that the EB/plant–magnetite residual solution chemistry was changed due to EB adsorption on ARM–Fe_3_O_4_, ROS–Fe_3_O_4_, MAT–Fe_3_O_4_, and JUN–Fe_3_O_4_ surfaces. The newly appeared peaks correspond to not attached (residual) SO^−^_3_, phenol, aniline, and phenyl groups, and the ring skeleton of benzene groups. More details about the identification of these peaks in EB/plant–magnetite residual solutions will be given in the next paragraphs, for the purpose of investigating the preferential attachment of the EB chromophore and auxochrome groups on all four magnetite surfaces.

##### 3.6.2.1 Analysis of preferential attachment of chromophore groups in EB adsorption

The spectra of all four EB/plant–magnetite residual solutions (Fig. 17) show that the NH bond peak of not attached phenyldiazonium groups shifted from 3387.56 in the EB aqueous reference solution to around 3302 cm^−1^, with however different peak bond areas. Additionally, new peaks, with different areas, appear at around 1634 cm^−1^ which correspond to the vibration of C=C of phenyl groups.^67^ Furthermore, the peaks of the ring skeleton of benzene groups appear, with different areas, in all four FTIR spectra at around 642 cm^−1^.^21^ It is worth noting that peak areas of NH of phenyldiazonium, C=C of phenyl, and CH of benzene groups vary in the same manner, where the peak area in the FTIR spectrum of EB/ARM–Fe_3_O_4_ is the narrowest one, followed by that of EB/ROS–Fe_3_O_4_, then that of EB/MAT–Fe_3_O_4_, and finally, the broadest one is that of EB/JUN–Fe_3_O_4_. This indicates that phenyldiazonium, phenyl, and benzene groups were more attached on ARM–Fe_3_O_4_, next on ROS–Fe_3_O_4_, then on MAT–Fe_3_O_4_, and finally, on the JUN–Fe_3_O_4_ surface.

Fig. 17 shows that the peak of the C≡C bond appears with different peak areas in the FTIR spectra of EB/MAT–Fe_3_O_4_ and EB/JUN–Fe_3_O_4_ (in the FTIR spectrum of EB/JUN–Fe_3_O_4_ it is broader than that of EB/MAT–Fe_3_O_4_ and attached on MAT–Fe_3_O_4_ more than in JUN–Fe_3_O_4_). In contrast, no peak corresponding to the C≡C bond appears in the FTIR spectra of EB/ROS–Fe_3_O_4_ and EB/ARM–Fe_3_O_4_ which confirms that C≡C bonds were completely attached on ROS–Fe_3_O_4_ and ARM–Fe_3_O_4_ surfaces, and almost completely attached on MAT–Fe_3_O_4_; however, these groups were less attached on the JUN–Fe_3_O_4_ surface.

Furthermore, the spectrum of EB/JUN–Fe_3_O_4_ shows minor peaks of N=N, C=C, and C=N (linked to the benzene ring) groups, whereas no peak corresponding to them appear in other FTIR spectra. This reveals that these groups were completely attached on MAT–Fe_3_O_4_, ROS–Fe_3_O_4_, and ARM–Fe_3_O_4_; however, these groups were less attached on the JUN–Fe_3_O_4_ surface.

It is also remarked that new peaks of the CH bond of CH_3_ of toluene groups appear at around 2343.82 cm^−1^^68^ in the FTIR spectrum of EB/JUN–Fe_3_O_4_ and a minor peak of toluene groups appears in the FTIR spectrum of EB/MAT–Fe_3_O_4_. Meanwhile, no peak corresponding to toluene groups appears in the FTIR spectra of EB/ARM–Fe_3_O_4_ and EB/ROS–Fe_3_O_4_. This reveals that toluene groups were completely attached on ROS–Fe_3_O_4_ and ARM–Fe_3_O_4_, and almost completely attached on MAT–Fe_3_O_4_; however, these groups were less attached on the JUN–Fe_3_O_4_ surface.

**Fig. 18:**
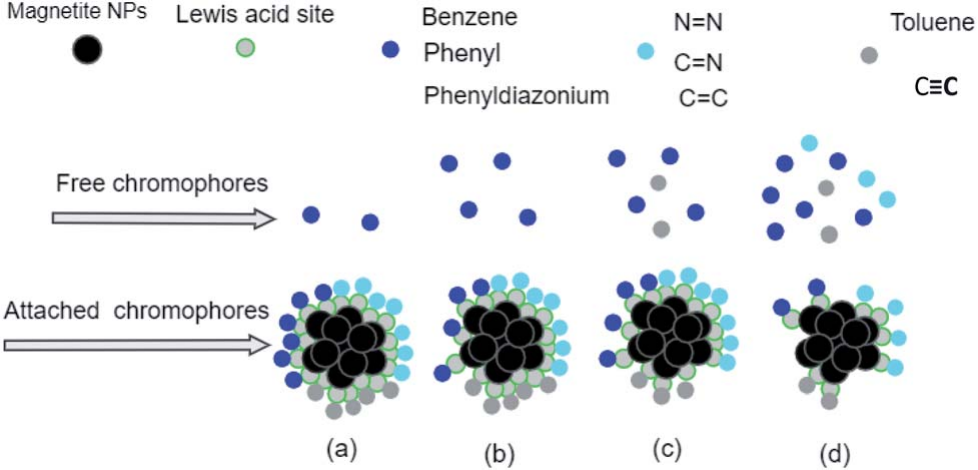
Preferential attachment of EB chromophore groups on (a) ARM–Fe_3_O_4_, (b) ROS–Fe_3_O_4_, (c) MAT–Fe_3_O_4_, and (d) JUN–Fe_3_O_4_ surfaces. The chromophore groups are grouped into three categories according to their behaviour in the attachment on the magnetite surface.

The analysis of preferential attachment of chromophore groups on magnetite surfaces shows, as summarized in Fig. 18, that:

• On JUN–Fe_3_O_4_, all EB chromophore groups were less attached compared to on the three other magnetite NPs.

• On MAT–Fe_3_O_4_, N=N, C=C, and C=N were completely attached, whereas C≡C and toluene groups were almost completely attached while other groups were more attached compared to on JUN–Fe_3_O_4_ and less attached compared to on ARM–Fe_3_O_4_ and ROS–Fe_3_O_4_.

• On ROS–Fe_3_O_4_, N=N, C=C, C=N, toluene, and C≡C were completely attached, while other groups were less attached than on ARM–Fe_3_O_4_ and more attached than on MAT–Fe_3_O_4_ and JUN–Fe_3_O_4_.

• On ARM–Fe_3_O_4_, N=N, C=C, C=N, toluene, and C≡C were completely attached, while phenyldiazonium, phenyl, and benzene groups were more attached than on other magnetite surfaces.

As it is known that chromophore groups prefer to attach to Lewis acid sites, it is possible to infer that the density of Lewis acid sites of ARM–Fe_3_O_4_ is the highest one, followed by that of ROS–Fe_3_O_4_, then that of MAT–Fe_3_O_4_, and finally that of JUN–Fe_3_O_4_.

The functional group attachment analysis results are consistent with EB adsorption yields, being the highest on ARM–Fe_3_O_4_ (86.05%), then on ROS–Fe_3_O_4_ (77.71%), next on MAT–Fe_3_O_4_ (61.98%), and finally on JUN–Fe_3_O_4_ (56.88%). This is due to the fact that most of the EB functional groups are chromophores.

##### 3.6.2.2 Analysis of preferential attachment of auxochrome groups in EB adsorption

FTIR spectra of EB/ARM–Fe_3_O_4_ and EB/ROS–Fe_3_O_4_ show that new peaks corresponding to SO^−^_3_,^62^ to the bending vibration of OH of phenol^69^ and to the bending vibration of NH of aniline groups^70^ appear at 1099.04 and 1204.58 cm^−1^, respectively (Fig. 17). In the FTIR spectrum of EB/ARM–Fe_3_O_4_, this peak is slightly more intense than in that of EB/ROS–Fe_3_O_4_. Meanwhile, no peaks corresponding to these groups appear in the FTIR spectra of EB/MAT–Fe_3_O_4_ and EB/JUN–Fe_3_O_4_ (Fig. 17).

The analysis of preferential attachment of auxochrome groups shows that:

• On ARM–Fe_3_O_4_, all EB auxochrome groups were less attached compared to on the three other magnetite NPs.

• On ROS–Fe_3_O_4_, sulphonic acid, phenol, and aniline groups were almost completely attached.

• On MAT–Fe_3_O_4_, all auxochrome groups were completely attached.

• On JUN–Fe_3_O_4_, all auxochrome groups were completely attached.

This leads to the conclusion that the densities of Brønsted acid sites of JUN–Fe_3_O_4_ and MAT–Fe_3_O_4_ surfaces are higher than those of ARM–Fe_3_O_4_ and ROS–Fe_3_O_4_ surfaces.

#### 3.6.3 The effect of the mediating plant extract's acidity on magnetite acid sites and adsorption yields of MO and EB azo dyes

Results in Table 11 showed that adsorption yields and capacities of both EB and MO differed on magnetite samples because of the variation of mediating plant extracts. Indeed, both EB and MO were highly adsorbed on ARM–Fe_3_O_4_, next on ROS–Fe_3_O_4_, then on MAT–Fe_3_O_4_, and finally on the JUN–Fe_3_O_4_ surface. These finding are in conformity with the results of several studies^16,28,29^ witch reported that the variation of mediating plant extract has a clear effect on the greenly synthesised adsorbent surface reactivity in dye adsorption yield and capacity. As it is known that dye adsorption is directly affected by the reactivity of the adsorbent surface towards the attachment of dyes' functional groups, the focus here is on the effect of the mediating plant extract's acidity on the preferential attachment of EB and MO dye functional groups in their adsorption processes on the greenly synthesized magnetite samples. To the best of our knowledge, this is the first report in which such a study has been conducted.

The analysis of the preferential attachment of chromophore and auxochrome groups in EB and MO adsorption leads to the conclusion that the Lewis acid site density is the highest on ARM–Fe_3_O_4_, next on ROS–Fe_3_O_4_, then on MAT–Fe_3_O_4_, and finally on the JUN–Fe_3_O_4_ surface. Moreover, EB and MO adsorption yields and capacities were highest on ARM–Fe_3_O_4_ (86.05%, 25.39 mg g^−1^ and 70.31%, 20.82 mg g^−1^, respectively), next on ROS–Fe_3_O_4_ (77.71%, 22.92 mg g^−1^ and 66.88%, 19.80 mg g^−1^), then on MAT–Fe_3_O_4_ (61.98%, 18.03 mg g^−1^ and 49.94%, 14.78 mg g^−1^), and finally on JUN–Fe_3_O_4_ (56.88%, 16.83 mg g^−1^ and 39.61%, 11.73 mg g^−1^), respectively. Accordingly, the adsorption yields and Lewis acid site densities varied in the same manner. Seeing that plant extracts used in the synthesis of ARM–Fe_3_O_4_, ROS–Fe_3_O_4_, MAT–Fe_3_O_4_ and –Fe_3_O_4_ NPs have respectively pH 5.25, 5.05, 4.63, and 3.69, one can conclude that plant extract pH has a clear effect on the preferential attachment of dye chromophore and auxochrome groups, magnetite nanoparticle acid sites, and adsorption yields. Indeed, the decrease of the mediating plant extract's acidity leads to the increase of Lewis acid site densities and the decrease of Brønsted acid site densities on magnetite NPs and hence an increase in the attachment of chromophore groups and a decrease in the attachment of auxochrome groups of dyes. As most of the MO and EB functional groups are chromophores, the decrease of the mediating plant extract's acidity also leads to an increase in adsorption yields. The remarked difference in adsorption yields of EB and MO on all four magnetite NPs is due to the fact that the ratio of chromophore/auxochrome groups in EB is remarkably greater than that in MO.

Thus, the plant extract's acidity could provide a preconceived idea about the densities of Brønsted and Lewis acid sites of magnetite NPs to be greenly synthesized and therefore about azo dye adsorption yields. Dye adsorption yield can be predicted according to the content of chromophore and auxochrome groups in the azo dye structure.

### 3.7 Desorption efficiency of dyes from magnetite surfaces and their stability after 3 cycles of reuse

#### 3.7.1 Desorption of dyes from magnetite surfaces

Desorption yields of EB and MO from magnetite surfaces (adsorbed at pH = 4) in the pH range of 8–12 are calculated using eqn (13) and they are shown in Table 12 and Fig. 20. It was observed that with the increase in solution pH desorption, EB and MO desorption efficiencies increase. At pH = 8, the desorption efficiencies of EB on ARM–Fe_3_O_4_, ROS–Fe_3_O_4_, MAT–Fe_3_O_4_ and JUN–Fe_3_O_4_ NPs were respectively 69.33, 64.77, 61.69, and 59.78%, where desorption efficiencies of MO on ARM–Fe_3_O_4_, ROS–Fe_3_O_4_, MAT–Fe_3_O_4_ and JUN–Fe_3_O_4_ NPs were respectively 62.83, 61.71, 60.31, and 57.88%. However, at higher pH (pH = 12), almost 100% desorption was achieved from the magnetite surfaces in each case. This is mainly due to the electrostatic repulsion between the negatively charged functional groups of EB and MO molecules and the negatively charged active sites of magnetite surfaces.

**Table 12:** Desorption yields of EB and MO from magnetite surfaces in different pH solutions after 60 minutes. Standard deviation of three replicates is mentioned

Sample	pH	EB des. (%)	STD	MO des. (%)	STD
JUN–Fe_3_O_4_	8	59.35	1.65	57.91	1.71
MAT–Fe_3_O_4_	8	61.35	1.83	60.38	1.69
ROS–Fe_3_O_4_	8	64.62	1.75	61.72	1.83
ARM–Fe_3_O_4_	8	69.05	1.88	62.05	1.59
JUN–Fe_3_O_4_	9	73.25	1.67	69.45	1.62
MAT–Fe_3_O_4_	9	74.15	1.78	72.74	1.57
ROS–Fe_3_O_4_	9	77.42	1.70	75.42	1.61
ARM–Fe_3_O_4_	9	81.15	1.62	79.05	1.85
JUN–Fe_3_O_4_	10	80.73	1.78	78.88	1.96
MAT–Fe_3_O_4_	10	81.25	1.69	80.82	1.55
ROS–Fe_3_O_4_	10	84.44	1.65	81.84	1.78
ARM–Fe_3_O_4_	10	89.09	1.57	84.79	1.77
JUN–Fe_3_O_4_	11	91.05	1.81	85.75	1.79
MAT–Fe_3_O_4_	11	92.19	1.68	88.11	1.74
ROS–Fe_3_O_4_	11	95.77	1.59	89.01	1.85
ARM–Fe_3_O_4_	11	97.10	1.49	93.90	1.87
JUN–Fe_3_O_4_	12	100.0	1.89	100.0	1.71
MAT–Fe_3_O_4_	12	100.0	1.59	100.0	1.61
ROS–Fe_3_O_4_	12	100.0	1.55	100.0	1.88
ARM–Fe_3_O_4_	12	100.0	1.54	100.0	1.63

#### 3.7.2 The stability of magnetite samples after 3 cycles of reuse

**Fig. 19:**
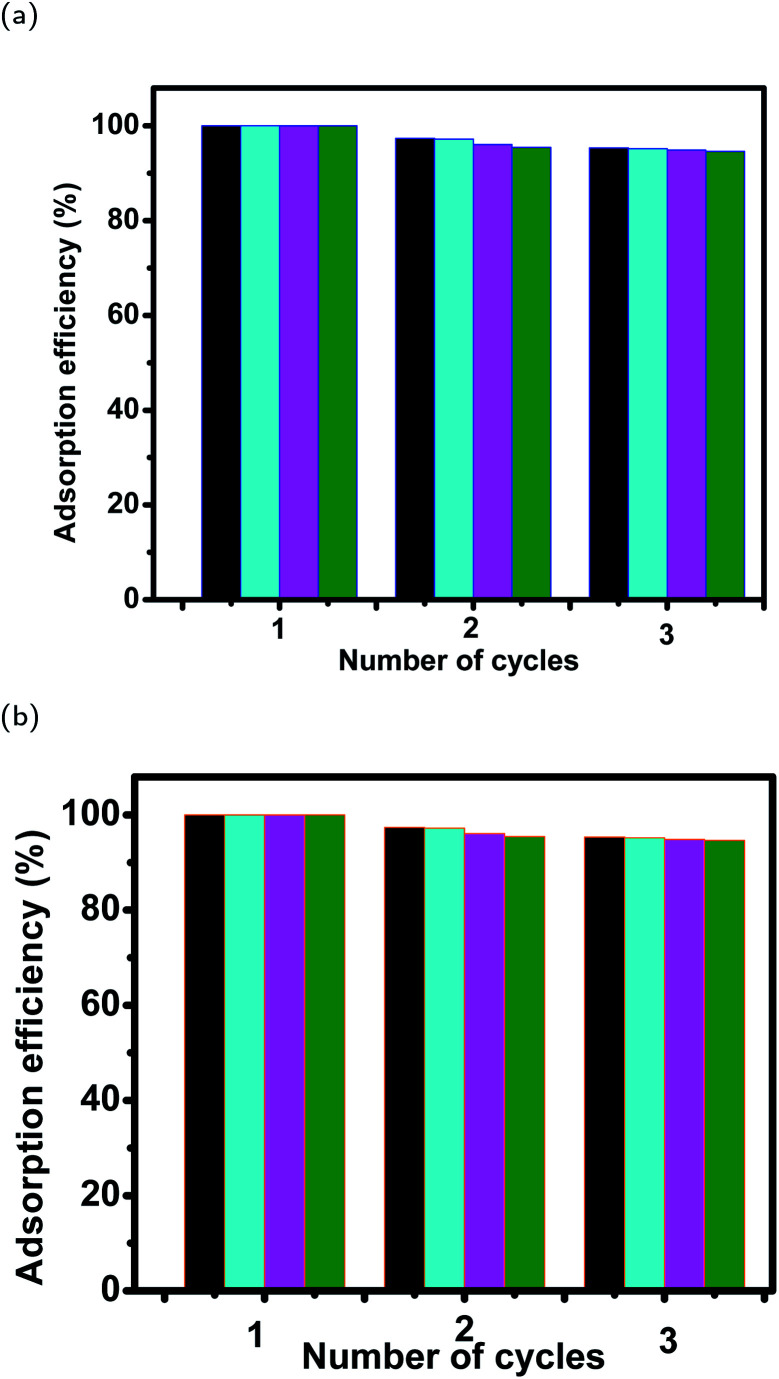
Stability of magnetite samples in (a) EB and (b) MO adsorption experiments after 3 cycles of reuse.

The multi-cycle efficiencies of magnetite samples were also tested for each case to evaluate their stability during dye adsorption experiments. The relative adsorption efficiencies of magnetite samples for MO and EB dyes were significant (97–95% and 94–91%) after the second and third cycles of reuse, where the average adsorption efficiencies of the first cycle were found to be 100% in all plant–Fe_3_O_4_ samples (see Fig. 19). This indicates the stability of all plant–Fe_3_O_4_ samples during EB and MO adsorption experiments.

**Fig. 20:**
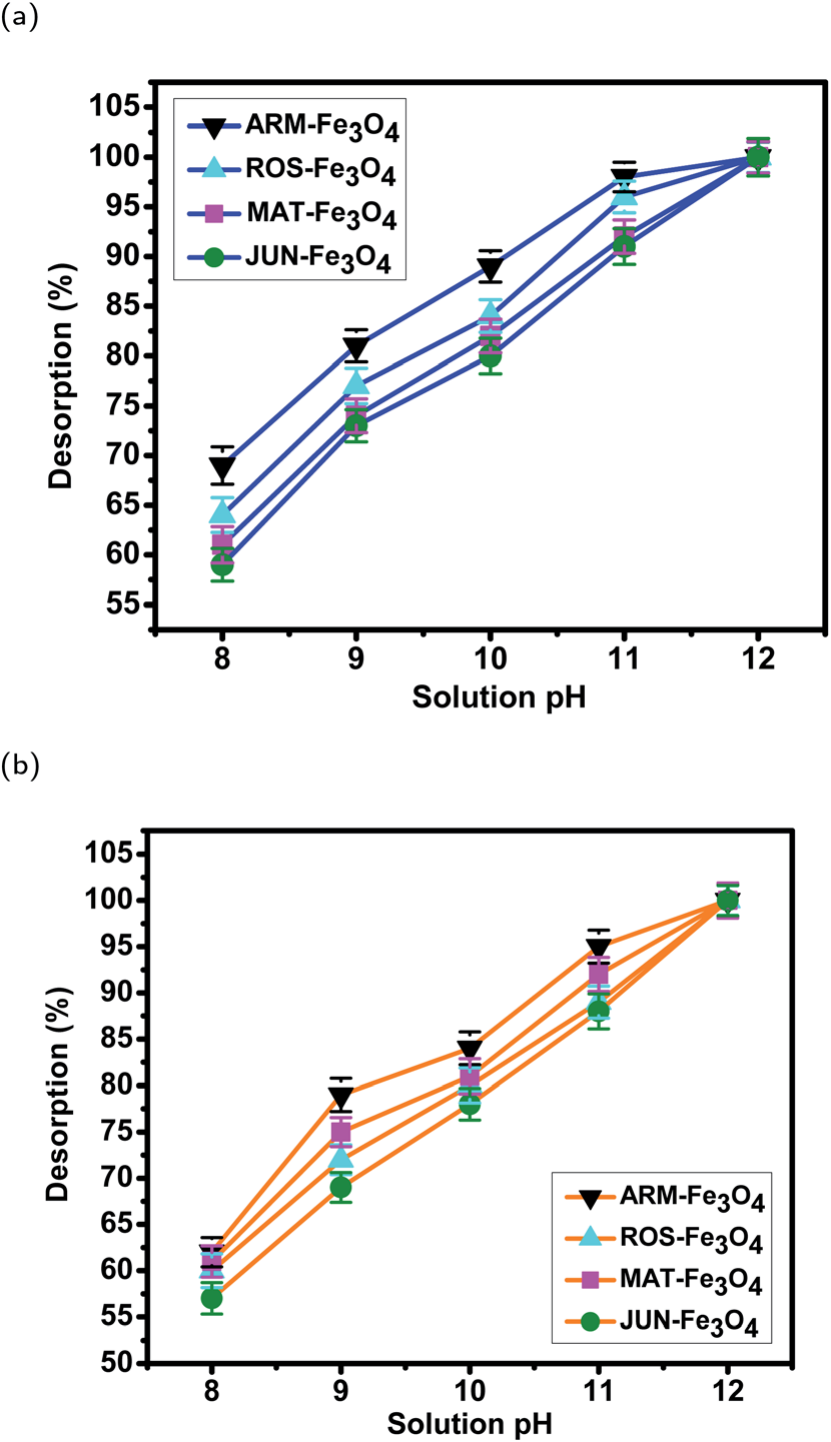
Desorption efficiency of (a) EB and (b) MO from magnetite samples in different pH solutions. Error bars represent the standard deviation of three replicates.

## 4 Conclusion

The adsorption of methyl orange (MO) and Evans blue (EB) azo dyes on four greenly synthesized magnetite NPs has been studied. The pHs of plant extracts used for the green synthesis were 5.25, 5.05, 4.63, and 3.69. The aim of the study was the investigation of the plant extract's acidity effect on the magnetite surface reactivity through the analysis of the preferential attachment of the dyes' chromophore and auxochrome groups on magnetite nanoparticles, and adsorption yields, and therefore determination of the plant extract pH effect on acid site types and densities. To do so, the free chromophore and auxochrome groups that are not attached to magnetite surfaces have been deeply analyzed in all dye residual solutions using FTIR spectroscopy, so as to perceive preferential attachment of dye chromophore and auxochrome groups on the four magnetite surfaces.

Obtained results show that the mediating plant extract's acidity has a clear effect on preferential attachment of dye chromophore and auxochrome groups, magnetite nanoparticle acid sites, and adsorption yields. Indeed, the decrease of plant extract acidity leads to the increase of Lewis acid site densities and the decrease of Brønsted acid site densities on magnetite NPs and hence an increase in the attachment of chromophore dye groups and a decrease in the attachment of auxochrome dye groups. As most of the MO and EB functional groups are chromophores, the decrease of the mediating plant extract's acidity also leads to an increase in adsorption yields.

The linear and non-linear pseudo-first-order and pseudo-second-order kinetics of the adsorption as well as the intra-particle diffusion mechanism have also been analyzed. Obtained results indicated that the adsorption kinetic followed a linear pseudo-first-order kinetic model. Meanwhile, film diffusion was found to be the step that controlled the adsorption mechanism of MO and EB adsorption processes. The thermodynamic studies of EB and MO adsorption processes have been analyzed in the temperature range of 303.15–318.15 K. They reveal the physical and endothermic nature of the adsorption in all cases.

## Author contributions

Kaouthar Ahmouda: writing – original draft, resources, investigation, visualization, methodology, and revision. Moussa Boudiaf: visualization and revision. Boubaker Benhaoua: visualization.

## Conflicts of interest

There are no conflicts to declare.

## Supplementary Material
