# Correction: A novel study on the preferential attachment of chromophore and auxochrome groups in azo dye adsorption on different greenly synthesized magnetite nanoparticles: investigation of the influence of the mediating plant extract's acidity

**DOI:** 10.1039/d3na90121a

**Published:** 2024-01-04

**Authors:** Kaouthar Ahmouda, Moussa Boudiaf, Boubaker Benhaoua

**Affiliations:** a Department of Process Engineering and Petrochemistry, Faculty of Technology, University of El Oued El Oued 39000 Algeria ahmouda-kaouthar@univ-eloued.dz drssmoussa@yahoo.fr; b Renewable Energy Research Unit in Arid Zones, University of El Oued El Oued 39000 Algeria; c LCIMN, Laboratory, Department of Process Engineering, Faculty of Technology, University Ferhat Abbas Setif 19000 Sétif Algeria benhaouab@yahoo.fr; d Department of Physics, Faculty of Exact Sciences, University of El Oued El Oued 39000 Algeria

## Abstract

Correction for ‘A novel study on the preferential attachment of chromophore and auxochrome groups in azo dye adsorption on different greenly synthesized magnetite nanoparticles: investigation of the influence of the mediating plant extract's acidity’ by Kaouthar Ahmouda *et al.*, *Nanoscale Adv.*, 2022, **4**, 3250–3271, DOI: https://doi.org/10.1039/D2NA00302C.

The authors apologise that it was not made clear that the synthesis and characterization of the greenly synthesized magnetite NPs used were previously disclosed in ref. 30 of the article.

Fig. 2–6 were previously published in ref. 30, and there are portions of text overlap in the Introduction and Materials and methods sections, and sub-sections 3.1–3.5 of the Results and discussion section.

Due to the absence of a separate publication dedicated to the synthesis results, the authors included the synthesis process and characterization in both articles, without due acknowledgment, leading to overlap in both the text and images used. While the method is similar, the kinetics of reactions in the two papers are entirely different.

The Royal Society of Chemistry apologises for these errors and any consequent inconvenience to authors and readers.

## Supplementary Material

